# Inhibition of NK1.1 signaling attenuates pressure overload-induced heart failure, and consequent pulmonary inflammation and remodeling

**DOI:** 10.3389/fimmu.2023.1215855

**Published:** 2023-07-24

**Authors:** Xiaochen He, Rui Xu, Lihong Pan, Umesh Bhattarai, Xiaoguang Liu, Heng Zeng, Jian-Xiong Chen, Michael E. Hall, Yingjie Chen

**Affiliations:** ^1^ Department of Physiology and Biophysics, University of Mississippi Medical Center, School of Medicine, Jackson, MS, United States; ^2^ College of Sports and Health, Guangzhou Sport University, Guangzhou, China; ^3^ Department of Pharmacology and Toxicology, University of Mississippi Medical Center, School of Medicine, Jackson, MS, United States; ^4^ Department of Medicine, University of Mississippi Medical Center, School of Medicine, Jackson, MS, United States

**Keywords:** NK1.1, pressure overload, IFN-γ, inflammation, heart failure

## Abstract

**Background:**

Inflammation contributes to heart failure (HF) development, the progression from left ventricular failure to pulmonary remodeling, and the consequent right ventricular hypertrophy and failure. NK1.1 plays a critical role in Natural killer (NK) and NK T (NKT) cells, but the role of NK1.1 in HF development and progression is unknown.

**Methods:**

We studied the effects of NK1.1 inhibition on transverse aortic constriction (TAC)-induced cardiopulmonary inflammation, HF development, and HF progression in immunocompetent male mice of C57BL/6J background.

**Results:**

We found that NK1.1^+^ cell-derived interferon gamma^+^ (IFN-γ^+^) was significantly increased in pulmonary tissues after HF. In addition, anti-NK1.1 antibodies simultaneously abolished both NK1.1^+^ cells, including the NK1.1^+^NK and NK1.1^+^NKT cells in peripheral blood, spleen, and lung tissues, but had no effect on cardiopulmonary structure and function under control conditions. However, systemic inhibition of NK1.1 signaling by anti-NK1.1 antibodies significantly rescued mice from TAC-induced left ventricular inflammation, fibrosis, and failure. Inhibition of NK1.1 signaling also significantly attenuated TAC-induced pulmonary leukocyte infiltration, fibrosis, vessel remodeling, and consequent right ventricular hypertrophy. Moreover, inhibition of NK1.1 signaling significantly reduced TAC-induced pulmonary macrophage and dendritic cell infiltration and activation.

**Conclusions:**

Our data suggest that inhibition of NK1.1 signaling is effective in attenuating systolic overload-induced cardiac fibrosis, dysfunction, and consequent pulmonary remodeling in immunocompetent mice through modulating the cardiopulmonary inflammatory response.

## Introduction

Left ventricular (LV) failure or heart failure (HF) is one of the major public health problems worldwide (1). Chronic systolic pressure overload induced by hypertension or aortic stenosis often results in cardiac hypertrophy, fibrosis, and LV failure. Chronic LV failure can then cause pulmonary inflammation, vascular remodeling, pulmonary hypertension (PH), right ventricular (RV) hypertrophy, and RV failure (2). The transitional process from LV failure to PH and RV failure will be termed as HF progression (3–6). LV failure-induced PH is the most common PH in clinics, and HF patients with PH have poor clinical outcomes (7).

Inflammation exerts an important role in the development of various cardiopulmonary diseases, such as atherosclerosis and HF development (8–12). Cardiac inflammation not only promotes cardiomyocyte injury, fibrosis, and LV failure, but it also promotes pulmonary inflammation and remodeling through modulating LV failure. Pulmonary inflammation also directly promotes lung injury, fibrosis, vessel remodeling, and the consequent RV failure (3–6). Previous studies have demonstrated the important roles of interferon-gamma (IFN-γ), tumor necrosis factor-α (TNF-α), interleukin-1beta (IL-1β), and IL6 in cardiac hypertrophy and dysfunction (12–16). Recent studies from us and others have demonstrated that cardiac inflammation is mediated by immune cells such as macrophages, CD4^+^ T cells, and CD11c^+^ antigen-presenting cells (5, 16–20). However, more research is needed to fully understand the roles of different immune cells, particularly lymphocytes, in HF development and progression.

NK1.1 plays an important role in Natural killer (NK) and natural killer T (NKT) cells, two cytotoxic lymphocyte subsets that exert critical roles in both innate and acquired immunity in tissue injury repair, maintaining tissue homeostasis against tumor growth, and bacterial and viral infections (21). NK1.1^+^NK and NK1.1^+^NKT cells also regulate immunity through an interaction with other immune cells, either by direct contact-mediated cytotoxicity and lysis of macrophages (Mφ), dendritic cells (DCs), and T cells through perforin and granzyme dependent fashion, or by releasing pro-inflammatory cytokines such as IFN-γ, a dimerized soluble cytokine that is the only member of the type II class of interferons (22). For example, NK cells promote the maturation and activation of Mφ and DCs partially by releasing IFN-γ (22). NK cells are predominantly derived from hematopoietic stem cells in the bone marrow and then migrate to peripheral tissues, but NK progenitors and immature NK cells may mature in secondary lymphoid tissues (23)

Interestingly, recent studies showed that NK and NKT cells play important roles in regulating the development of idiopathic PH (24–26). For example, an earlier study showed that impairment of NK cells was associated with idiopathic PH in patients (25). In addition, both Nfil3 gene knockout mice and NKp46-GFP mice, two mouse strains with NK cell deficiency (27, 28), developed spontaneous PH in mice (26). In addition, a recent study showed that pulmonary vessel fibrosis in samples from idiopathic pulmonary fibrosis patients was associated with decreased NKT cells, while pharmacological NKT cell activation using a synthetic α-galactosylceramide analog, KRN7000, restored local NKT cell numbers and ameliorated vascular remodeling and PH in bleomycin-induced lung fibrosis in mice (24), indicating NKT cell deficiency contributes to lung fibrosis-induced PH. Moreover, a previous study demonstrated that invariant NKT cell-deficient J*α*18 knockout (KO) mice are protected from TAC-induced cardiac inflammation and dysfunction (29). However, NKT cells mediate pulmonary inflammation and dysfunction in murine sickle cell disease (30), a mouse model showing increased PH. Furthermore, a recent study showed that transverse aortic constriction (TAC) resulted in a significant increase in NK cell infiltration in the LV in mice (31). These findings suggest that NK and NKT cells may potentially attenuate the development of other types of PH, such as HF-induced PH through modulating pulmonary inflammation, fibrosis, and vessel remodeling. Conversely, as IFN-γ promotes maturation and activation of Mφ and DCs, NK and NKT cells may exacerbate cardiac inflammation, HF development, and HF progression through modulation of IFN-γ signaling (32, 33). Nevertheless, the role of NK1.1 signaling in regulating cardiopulmonary inflammation, HF development, and HF progression has not previously been investigated.

In the present study, we examined the effect of systemic inhibition of NK1.1 on systolic overload-induced cardiac inflammation, hypertrophy, dysfunction, and the consequent lung remodeling and RV hypertrophy in immune-competent adult C57B6J mice. To our surprise, we found that inhibition of NK1.1 signaling using anti-NK1.1 antibodies significantly attenuated rather than exacerbated cardiac inflammation, HF development, and HF progression in these immunocompetent mice in response to TAC, a commonly used method generating systolic cardiac overload in experimental animals.

## Materials and methods

### Animals and experiment protocols

Male C57BL/6J mice purchased from The Jackson Laboratory (Bar Harbor, ME) were fed laboratory standard chow and water and housed in ventilated cages in a temperature-controlled facility with 12-hour light/dark cycles. All protocols were approved by the Institutional Animal Care and Use Committee at the University of Mississippi Medical Center.

Adult C57BL/6J mice were subjected to TAC or sham surgery. The mice after TAC or sham surgery were randomly assigned to groups treated with either anti-NK1.1 antibodies (clone PK136, Bioxcell, BE0036, 250 μg/mouse/dose) or IgG2α control isotype diluted in 100 μl phosphate-buffered saline (PBS) through intraperitoneal injection every third day, starting ten days after surgery. Mice were sacrificed at the end of the eight weeks after surgery. The dosage of the blocking antibodies was based on previous reports (34, 35), and our preliminary data that the above dosage of blocking antibodies effectively depleted NK and NKT cells in the peripheral blood and spleen of control mice (**Supplementary Figure S1**).

TAC Procedure: LV pressure overload was induced by TAC surgery for 8 weeks as previously described (36–38). Briefly, the mice were anesthetized with a single intraperitoneal injection of ketamine (100 mg/kg) and xylazine (10 mg/kg) (2). A blunt 27-gauge needle was used to determine the degree of aortic constriction induced by ligation. The sham procedure was identical, except that there was no ligation of the aorta. Mice were subcutaneously injected with Buprenorphine SR (0.1mg/kg) shortly before waking up.

### Echocardiography

Transthoracic echocardiograms were performed by using a Vevo 2100 high-resolution imaging platform equipped with an MS400 18–38 MHz linear array ultrasound transducer (FUJIFILM Visual Sonics Inc., Canada). The mice were anesthetized by inhalation of 1–2% isoflurane mixed with 100% medical oxygen. M-mode cine loops were recorded and analyzed by Vevo LAB software (FUJIFILM Visual Sonics Inc., Canada) to assess LV ejection fraction (EF), fractional shortening (FS), end-systolic diameter (LVESD), LV end-diastolic diameter (LVEDD), LV end-systolic volume (LVESV), LV end-diastolic volume (LVEDV), LV anterior wall and posterior wall thickness at end-systole or end-diastole, stroke volume, and cardiac output (4, 36, 37, 39, 40).

### Tissue collection and flow cytometry analysis

Mice were anesthetized with a single intraperitoneal injection of ketamine (100 mg/kg) and xylazine (10 mg/kg). The lung was perfused with cold PBS through the RV, and the lung tissues were digested in Hanks’ balanced salt solution (HBSS) containing 1mg/mL of collagenase D and 60U/mL of DNAse I (Millipore Sigma, MO) on the gentle MACS™ Octo dissociator. The cells were isolated and flow cytometry analysis was performed as previously described with minor modifications (5). Single-cell suspensions were stained with fixable viability dye (FVS440UV, BD Bioscience, CA) in PBS followed by washing in staining buffer and blocking with anti-mouse CD16/32 (clone 2.4G2, Biolegend, CA) antibodies to prevent non-specific binding of antibodies to FcRγ. The cells were then incubated with fluorescence-conjugated anti-CD45, CD3, CD4, CD8, CD19, NK1.1, CD11b, CD11c, Ly6C, Ly6G, F4/80, CD44, CD62L, and major histocompatibility complex class II molecule (MHC-II) antibodies (**Supplementary Table S1**), accordingly. For cytokine production analysis, single cell suspensions were stimulated with a cell stimulation cocktail (eBioscience, MA) diluted in RPMI medium containing 10% fetal bovine serum (FBS) for two hours in a cell culture incubator at 37°C with 5% CO_2_. The cells were then collected and stained with FVS440UV, blocked by anti-mouse CD16/32, and labelled with fluorescence-conjugated anti-CD45, CD3, CD4, CD8, F4/80, NK1.1, IFN-γ, IL-1β, TNF-α, programmed death-1 (PD-1), Ly6G, CD69, perforin-1, or granzyme A (**Supplementary Table S1**). Samples were run on the BD FACSymphony™ A3 Cell Analyzer (BD Biosciences, CA). Data were analyzed by FlowJo_V10 (FlowJo, OR) software.

### Multiplexed cytokine assay

Lung tissues were grounded to powder by mortar and pestle in liquid nitrogen and dissolved in PBS containing 1x Halt™ protease inhibitor cocktail (ThermoFisher Scientific, MA). The content of various cytokines in lung homogenates was determined by LEGENDplex™ (Mouse Inflammation panel, #740150, BioLegend) according to the manufacturer’s protocol.

### Histological analysis and immunofluorescence staining

Tissues were excised, fixed in 10% formalin, and then embedded in paraffin. 5-micron sections were stained with Sirius red and Fast green Stain Kit (Chondrex, WA) to evaluate LV, RV, and pulmonary fibrosis. Heart sections were also stained with Alexa Fluor 488-conjugated wheat germ agglutinin (Invitrogen, 5μg/ml) to evaluate LV and RV myocyte size. For immunofluorescence staining of CD45^+^ leukocytes, NK1.1^+^ cells, and macrophages, antigen retrieval was performed in heated citrate buffer (pH 6.0) and the sections were blocked with 1% bovine serum albumin in PBS for one hour at room temperature, followed by incubation with goat anti-CD45 (R&D systems, 1:100), mouse anti-NK1.1 (Bioxcell, 1: 100), or goat anti-MAC-2 (R&D systems, 1:200) primary antibodies at 4°C overnight. The tissue sections were then incubated with Alexa Fluor 555-conjugated secondary donkey anti-goat or goat anti-mouse (Invitrogen, 1:500), respectively. All images were captured using an Olympus BX43 fluorescent microscope, then quantified using the inForm Advanced Image Analysis (Akoya Biosciences, MA), and Image-J software.

### Statistical analysis

Data are presented as mean ± SEM. The assumptions of normality in both comparison groups were determined by the normality test. Statistical significance between the two groups was determined using a two-tailed Student’s t-test. A two-way ANOVA followed by a Bonferroni correction *post hoc* test was used to test the differences among control and TAC mice with or without depletion of NK1.1^+^ cells using GraphPad Prism 9 software. p <0.05 was considered statistically significant.

## Results

### Pulmonary NK1.1^+^ cells-derived IFN-γ production is increased after HF

**Figure 1:**
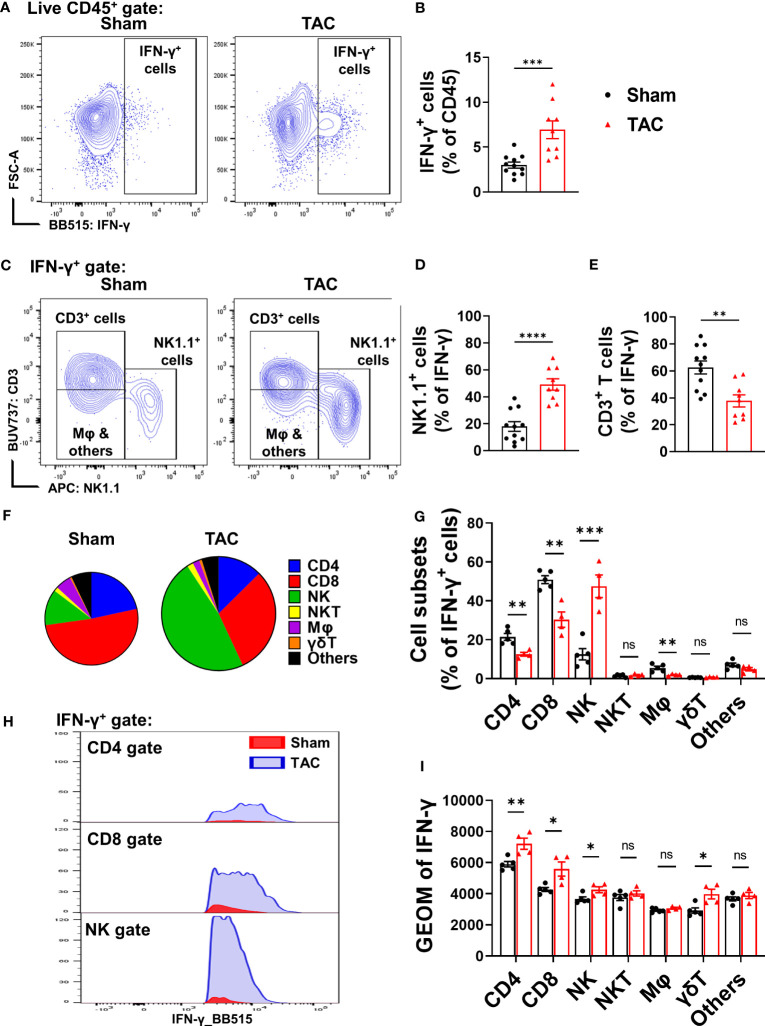
Heart failure (HF) is associated with increased pulmonary IFN-γ^+^ NK1.1^+^ cells. **(A, B)** Flow cytometry analysis of the lung tissue shows an increased percentage of IFN-γ^+^ cells in the CD45+ subset in the HF mice. n=9-11. **(C–E)** In the IFN-γ^+^ subset, T cells (CD3^+^) and natural killer (NK1.1^+^) cells are the major cell populations (n=9-11). **(F, G)** Changes of percentages of IFN-γ^+^ CD4 T cells, CD8 T cells, NK1.1^+^NK cells, F4/80 macrophage, γδ T cells, and other immune cells after HF (n=4-5). **(H, I)** the alterations of IFN-γ producing capacity in the CD4^+^ T cells, CD8^+^ T cells, NK1.1^+^NK cells, and γδ T cells, as indicated by the histogram and geometric mean fluorescent intensity (n=4-5). ns, not significant. *p<0.05, **p<0.01, ***p<0.001, ****p<0.0001.

To identify cell subsets that produce IFN-γ, freshly isolated pulmonary cells were stimulated with a PMA cocktail and then determined IFN-γ^+^ cells using flow cytometry. The percentage of lung IFN-γ^+^ leukocytes in CD45^+^ immune cells was significantly increased in TAC-induced HF mice as compared with control wild type mice (**Figures 1A, B**). By using the gating strategy presented in **Supplementary Figure S2A**, we found that pulmonary T cells (CD3^+^ T cells) and NK1.1^+^ cells (including both NK and NKT cells) account for ~60% and ~20% of pulmonary IFN-γ^+^ cells in wild type mice under control conditions, respectively (**Figures 1C-E**). Interestingly, pulmonary IFN-γ^+^NK1.1^+^ cells increased from 20% to 50% after HF (**Figures 1C, D**), indicating that NK1.1^+^ cells are the major IFN-γ producing leukocytes after developing HF. The IFN-γ^+^ leukocytes were further grouped as CD4^+^ T cells, CD8^+^ T cells, γδT cells, NK1.1^+^NK cells, NK1.1^+^NKT cells, F4/80^+^ macrophages, and other cells (**Figures 1F, G**). The percentage of NK1.1^+^NK cells in the pulmonary IFN-γ^+^ cell subset was significantly increased after HF, while the percentages of pulmonary CD4^+^, CD8^+^ T cells, and F4/80^+^ macrophages in the IFN-γ^+^ cells were significantly decreased after HF (**Figure 1G**). The pulmonary NK1.1^+^NK cells were ~20-fold higher than NK1.1^+^NKT cells after HF (**Figure 1G**). The percentages of NK1.1^+^NKT cells, macrophages, and other cells in the IFN-γ^+^ cells were not significantly changed after HF (**Figure 1G**). Moreover, geometric mean fluorescent (GEO mean) intensities of these IFN-γ^+^ cells were significantly increased in NK1.1^+^NK cells, CD4^+^ cells, CD8^+^ cells, and γδT cells, indicating the average IFN-γ-producing capacities in these lymphatic cell subsets were significantly increased after HF (**Figures 1H, I**).

In addition, by using a flow cytometry-based cytokine assay, we determined the pulmonary content of IFN-γ and several other cytokines in the sham control and HF mice. The data showed that pulmonary IFN-γ^+^ content was significantly increased in mice after HF (**Supplementary Figure S2B**). Moreover, pulmonary TNF-α, monocyte chemoattractant protein-1 (MCP-1), IFN-β, IL-1β, IL-6, granulocyte-macrophage colony-stimulating factor (GM-CSF), and IL-27 protein contents were also significantly increased in mice after HF (**Supplementary Figure S2B**).

### Depletion of NK1.1+ cells significantly attenuated TAC-induced LV failure and inflammation

**Figure 2:**
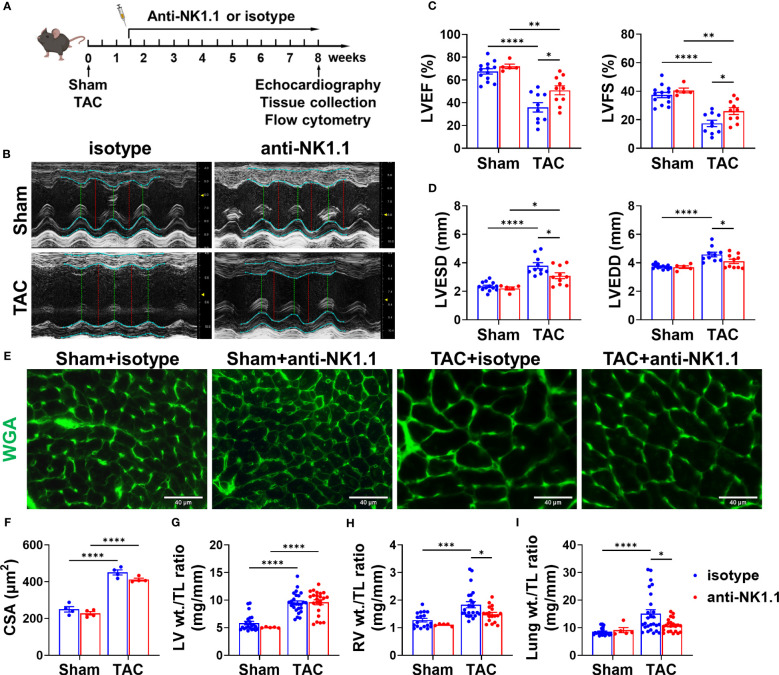
Depletion of NK1.1^+^ cells attenuated transverse aortic constriction (TAC)-induced left ventricle (LV) dysfunction in mice. **(A)** Schematic of experimental design for depletion of NK1.1^+^ cells with or without TAC-induced HF. **(B)** Representative images of echocardiography in the indicated groups. **(C)** Summarized echocardiography measurements of LV ejection fraction (LV EF), fractional shortening (LV FS). n=5-13. **(D)** Echocardiographic measurements of LV end-systolic diameter, and end-diastolic diameter. n=5-13. **(E, F)** Representative images of wheat germ agglutinin (WGA) staining and quantification of cardiomyocyte size in the indicated groups, showing that TAC-induced cardiomyocyte hypertrophy was not affected by depletion of NK1.1^+^ cells. **(G-I)** Tissue weight (LV, RV, and lung) to tibial length ratio. n=5-26. *p<0.05, **p<0.01, ***p<0.001, ****p<0.0001. CSA, cross-sectional area.

**Figure 3:**
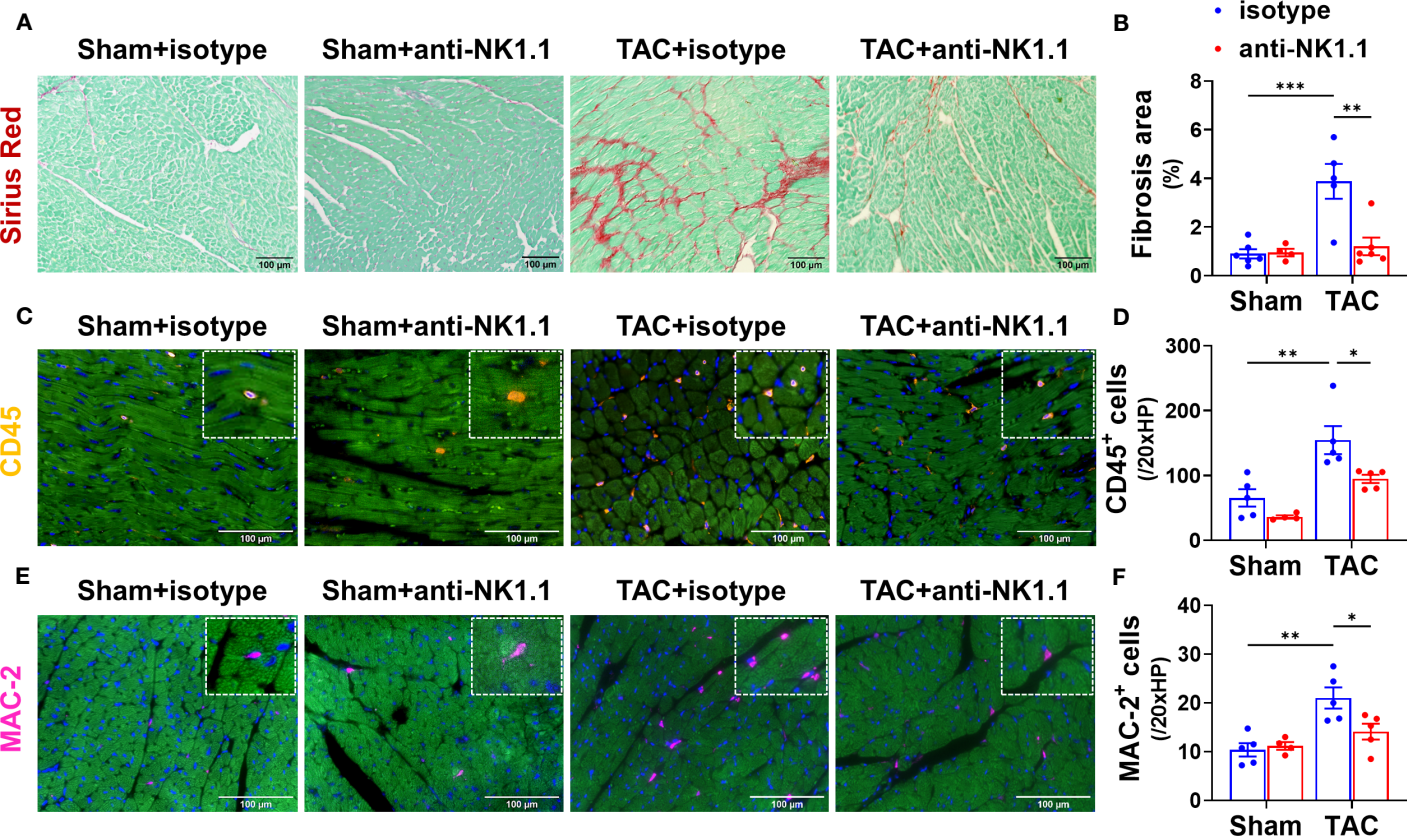
Depletion of NK1.1^+^ cells attenuated TAC-induced LV leukocyte infiltration and fibrosis in mice. **(A, B)** Representative images of Sirius Red staining and quantified LV interstitial fibrosis in the indicated groups. **(C, D)** Representative images of immunostaining of CD45 or MAC-2 and quantification of LV infiltration of CD45^+^ leukocytes in the indicated groups. **(E, F)** Representative images of immunostaining of LV MAC-2^+^ macrophage and quantification of the infiltration of CD45^+^ leukocytes or MAC-2^+^ macrophages. n=4-5. *p<0.05, **p<0.01, ***p<0.001.

Since pulmonary IFN-γ^+^NK1.1^+^ cells were significantly increased after HF, and since IFN-γ exerts an important role in HF development (32, 41), we examined the role of NK1.1^+^ cells on LV hypertrophy and dysfunction in control and HF mice by depleting NK1.1^+^ cells using anti-NK1.1 antibodies starting ten days after TAC surgery (**Figure 2A**). While systemic depletion of NK1.1^+^ cells had no detectable effect on LV structure and function in sham mice, depletion of NK1.1^+^ cells significantly attenuated TAC-induced reduction of LV EF and LV FS (**Figures 2B, C**). Depletion of NK1.1^+^ cells also significantly attenuated TAC-induced increases of LVESD and LVEDD (**Figure 2D**). Depletion of NK1.1^+^ cells did not significantly affect TAC-induced LV hypertrophy, as evidenced by the unchanged ratio of LV weight to tibial length, LV wall thickness at end diastole, LV wall thickness at end systole, and unchanged cardiomyocyte hypertrophy (**Figures 2E-G** and **Supplementary Figure S3**). Other echocardiographic parameters are listed in **Supplementary Table S2**. However, depletion of NK1.1^+^ cells significantly attenuated TAC-induced RV hypertrophy and increase of lung weight, as evidenced by a significantly smaller increase of RV weight, lung weight, and their ratio to tibial length (**Figures 2H, I** and **Supplementary Table S3**). Moreover, as shown in **Figure 3**, depletion of NK1.1^+^ cells significantly attenuated TAC-induced LV interstitial fibrosis and infiltration of CD45^+^ leukocytes or MAC-2^+^ leukocytes.

### Depletion of NK1.1^+^ cells attenuated TAC-induced RV inflammation and hypertrophy in mice

**Figure 4:**
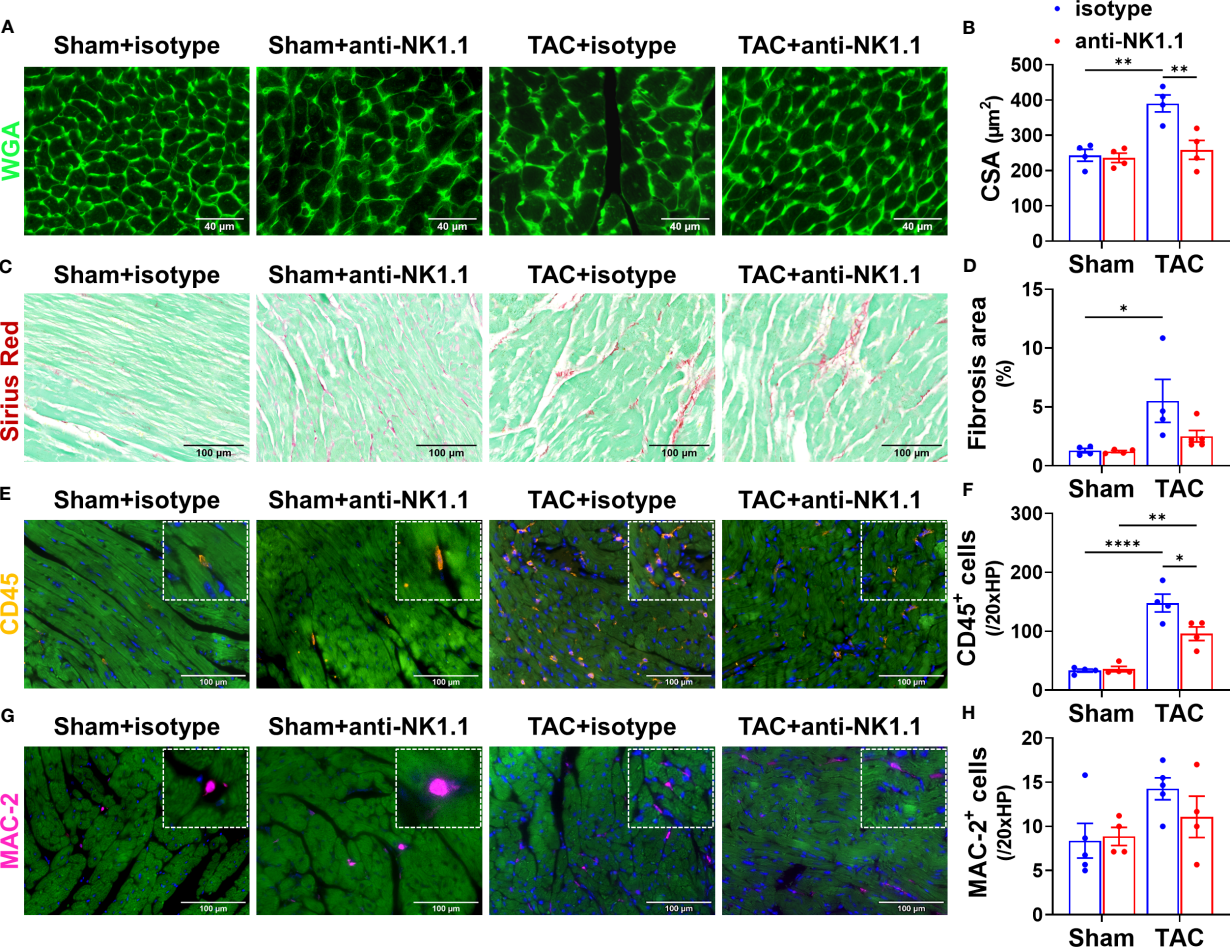
Depletion of NK1.1^+^ cells attenuated TAC-induced RV inflammation and hypertrophy in mice. **(A, B)** Representative images of WGA staining and quantification of cardiomyocyte size in the indicated groups, showing that TAC-induced RV hypertrophy was not attenuated by depletion of NK1.1^+^ cells. **(C, D)** Representative images of Sirius Red staining and quantification of interstitial fibrosis in the indicated groups in the RV. **(E, F)** Representative images of immunostaining of CD45 and quantification of the infiltration of CD45^+^ leukocytes in the RV of the indicated groups. **(G, H)** Representative images of immunostaining of MAC-2 and quantification of the infiltration of MAC-2^+^ macrophages in the RV of the indicated groups. n=4-5. *p<0.05, **p<0.01, ****p<0.0001. CSA, cross-sectional area.

Since RV hypertrophy and remodeling are important features of HF progression (3, 4), we further determined the effect of depletion of NK1.1^+^ cells on TAC-induced RV inflammation, cardiomyocyte hypertrophy, and fibrosis. Consistent with the decreased RV weight to tibial length ratios in mice after depletion of NK1.1^+^ cells, WGA staining demonstrated that depletion of NK1.1^+^ cells significantly attenuated TAC-induced increase of RV cardiomyocyte hypertrophy as indicated by the reduced cross-sectional area in the RV (**Figures 4A, B**). However, the depletion of NK1.1^+^ cells did not significantly affect TAC-induced RV fibrosis (**Figures 4C, D**). Depletion of NK1.1^+^ cells significantly attenuated TAC-induced RV leukocytes as evidenced by reduced infiltration of CD45^+^ cells (**Figures 4E, F**). However, TAC with or without depletion of NK1.1^+^ cells did not affect infiltration of MAC-2^+^ leukocytes in RV of these mice as compared with control isotype-treated TAC mice (**Figures 4G, H**).

### Depletion of NK1.1^+^ cells attenuated TAC-induced pulmonary inflammation, fibrosis, and vessel remodeling

**Figure 5:**
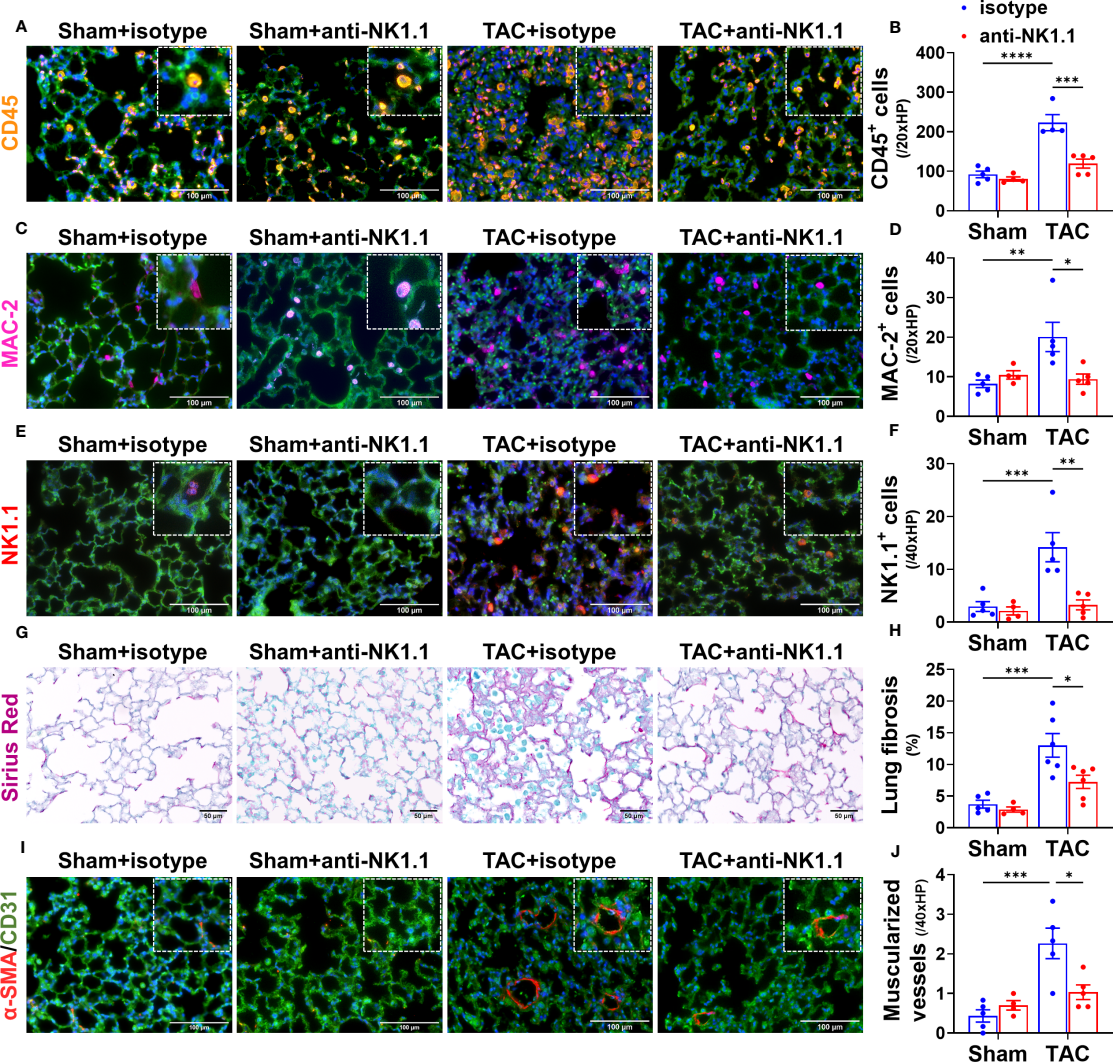
Depletion of NK1.1^+^ cells attenuated pulmonary leukocyte infiltration, fibrosis, and vascular remodeling. **(A-F)** Representative images of immunostaining of pulmonary CD45, MAC-2, or NK1.1 and quantified data in the indicated groups. **(G, H)** Representative images of Sirius Red staining and quantified interstitial fibrosis in the indicated groups in the lung. **(I, J)** Representative images of co-staining CD31 and α-smooth muscle actin (α-SMA) in the indicated groups, showing pulmonary vessel muscularization and remodeling. n=4-6. *p<0.05, **p<0.01, ***p<0.001, ****p<0.0001.

Since pulmonary inflammation exerts an important role in promoting pulmonary fibrosis, vessel remodeling, and RV hypertrophy under HF conditions (5, 6), we further determined the effect of systemic depletion of NK1.1^+^ cells on pulmonary leukocyte infiltration, fibrosis, and vessel remodeling. Depletion of NK1.1^+^ cells significantly reduced the number of infiltrating pulmonary CD45^+^ leukocytes and MAC-2^+^ leukocytes in TAC mice as compared with the corresponding IgG-treated TAC mice (**Figures 5A-D**). Pulmonary NK1.1^+^ cells in TAC mice were also significantly decreased as compared with control IgG-treated TAC mice (**Figures 5E, F**). Depletion of NK1.1^+^ cells also significantly reduced pulmonary interstitial fibrosis in the TAC mice (**Figures 5G, H**). Furthermore, the depletion of NK1.1^+^ cells significantly attenuated TAC-induced pulmonary vascular remodeling as evidenced by the alterations of pulmonary microvascular muscularization (**Figures 5I, J**).

### Depletion of NK1.1^+^ cells effectively reduced pulmonary IFN-γ^+^ NK and NKT cells

**Figure 6:**
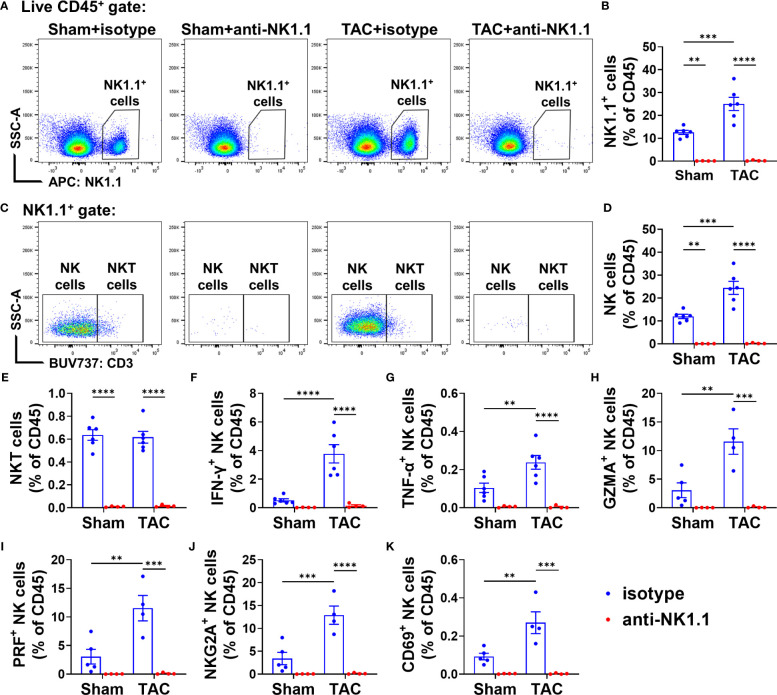
Anti-NK1.1 antibodies effectively depleted NK1.1^+^ cells and cytokine-producing NK1.1^+^NK and NK1.1^+^NKT cells. **(A, B)** Representative plots of flow cytometry and quantification of NK1.1^+^ cell populations in the indicated groups. **(C-E)** Representative plots of flow cytometry and quantification of the percentage of NK1.1^+^NK and NK1.1^+^NKT cells in the CD45^+^ cell subset. n=4-6. **(F-K)** Quantification of the percentage of IFN-γ^+^, TNF-α^+^, granzyme A^+^ (GZMA), CD69^+^, NKG2A^+^, or perforin^+^ (PRF) NK1.1^+^NK cells in the CD45^+^ cell subset. n=4-6. **p<0.01, ***p<0.001, ****p<0.0001.

We further determined the alterations of pulmonary NK1.1^+^NK cells, NK1.1^+^NKT cells, and their IFN-γ^+^ cell subsets in sham and TAC mice. As shown in **Figures 6A-E**, anti-NK1.1 blocking antibodies effectively depleted pulmonary NK1.1^+^NK and NK1.1^+^NKT cells in both TAC and sham mice. Pulmonary IFN-γ^+^NK1.1^+^NK and IFN-γ^+^NK1.1^+^NKT cells were also significantly attenuated in both sham and TAC mice after chronic administration of anti-NK1.1 blocking antibodies (**Figures 6F** and **Supplementary Figure S4A**). In addition, TNF-α^+^, perforin-1^+^, or granzyme-A^+^ NK1.1^+^NK and NK1.1^+^NKT cells were also significantly abolished in both sham and TAC mice after chronic administration of anti-NK1.1 blocking antibodies (**Figures 6G-I** and **Supplementary Figure S4A**).

Since the activation status of NK1.1^+^NK and NK1.1^+^NKT cells affect their function, we further determined their expressions of inhibitory molecule NKG2A and PD-1 (a common checkpoint inhibitor), and CD69, an activation marker. TAC caused significant increases of NKG2A^+^ NK1.1^+^NK or NK1.1^+^NKT cells in the CD45^+^ subset (**Figure 6J** and **Supplementary Figure S4B**). The percentage of PD1^+^ NK1.1^+^NK cells and PD1^+^ NK1.1^+^NKT cells was not significantly increased after TAC (**Supplementary Figures S4B, D**). In addition, TAC resulted in a significant increase in the percentage of CD69^+^ NK1.1^+^NK cells in the CD45^+^ subset, but it did not affect the percentage of CD69^+^ NK1.1^+^NKT cells in the CD45^+^ subset (**Figure 6K** and **Supplementary Figure S4B**). Moreover, as anticipated, the percentages of NKG2A^+^ or CD69^+^ NK1.1^+^NK cells or NK1.1^+^NKT cells in the CD45^+^ subset were abolished by depletion of NK1.1^+^ cells after TAC (**Figures 6J, K** and **Supplementary Figure S4D**). However, the percentages of NKG2A^+^, PD1^+^, CD69^+^ NK1.1^+^NK cells or NK1.1^+^NKT cells in total NK1.1^+^NK cells or NK1.1^+^NKT cells were largely unaffected by TAC (**Supplementary Figures S4C, E**).

### Effect of systemic depletion of NK1.1^+^ cells on pulmonary IFN-γ^+^ CD4^+^ and CD8^+^ T cell subsets

**Figure 7:**
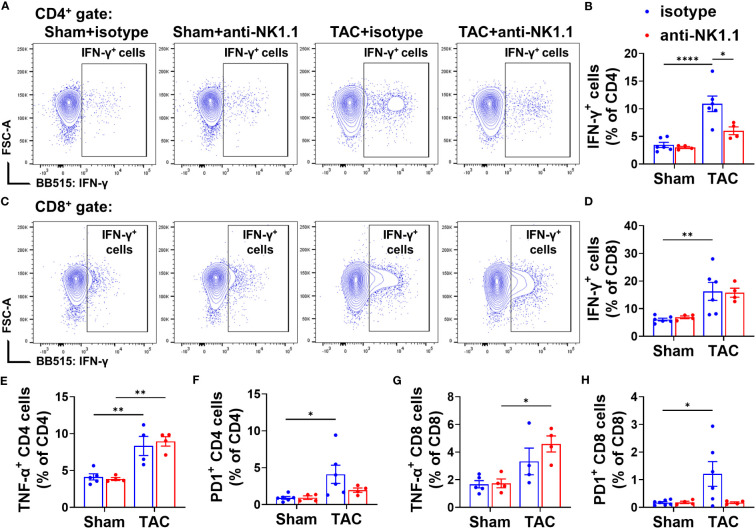
Effect of depletion of NK1.1^+^ cells on pulmonary IFN-γ^+^ CD4^+^ and CD8^+^ T cell subsets. **(A, B)** Representative images of flow cytometry plots of and relative percentage of IFN-γ^+^ cells in the CD4^+^ subset. **(C, D)** Representative images of flow cytometry plots of and relative percentage of IFN-γ^+^ cells in the CD8^+^ subset. **(E, F)** Relative percentage of TNF-α^+^ and PD1^+^ cells in the CD4^+^ subset. n=4-6. **(G, H)** Relative percentage of TNF-α^+^ and PD1^+^ cells in the CD8^+^ subset. n=4-6. *p<0.05, **p<0.01, ****p<0.0001.

Since NK1.1^+^ cells regulate immunity through an interaction with other immune cells, we further evaluated T cell activation in the lungs of these mice after TAC with or without NK1.1^+^ cell depletion. There was a small but significant decrease in the percentage of CD4^+^ T cells in the CD3^+^ population after TAC (**Supplementary Figures S5A, B**). Depletion of NK1.1^+^ cells did not change the percentage of CD4^+^ cells in the CD3^+^ population in mice after TAC, but it caused a significant increase in the percentage of the CD8^+^ cells in the CD3^+^ population (**Supplementary Figures S5B, C**). In addition, the expression of IFN-γ and TNF-α in CD4^+^ and CD8^+^ T cells was determined. The data showed that the percentages of IFN-γ^+^ cells in CD4^+^ and CD8^+^ T cell subsets were significantly increased in IgG-treated mice after TAC, and NK1.1^+^ cell depletion significantly attenuated TAC-induced increase of the percentage of IFN-γ^+^CD4^+^ cells in the CD4^+^ subset (**Figures 7A, B**). NK1.1^+^ cell depletion did not affect the percentage of IFN-γ^+^CD8^+^ cells in the CD8^+^ subset (**Figures 7C, D**). The percentage of TNF-α^+^ cells in CD4^+^ cell subset was significantly increased in mice after TAC (**Figure 7E**). NK1.1^+^ cell depletion did not affect the TAC-induced increase of the percentages of TNF-α^+^CD4^+^ cells in the CD4^+^ subset (**Figure 7E**), but it significantly increased the percentages of TNF-α^+^CD8^+^ cells in the CD8^+^ subset after TAC (**Figure 7G**). In addition, the expression of PD-1 was determined. The percentages of PD1^+^ cells in the CD4^+^ and CD8^+^ T cell subsets were significantly increased in IgG-treated mice after TAC (**Figures 7F, H**). Depletion of NK1.1^+^ cells showed a trend to decrease the percentages of PD1^+^ cells in CD4^+^ and CD8^+^ T cell subsets after TAC (**Figures 7F, H**).

### Depletion of NK1.1^+^ cells attenuated pulmonary T cell activation

**Figure 8:**
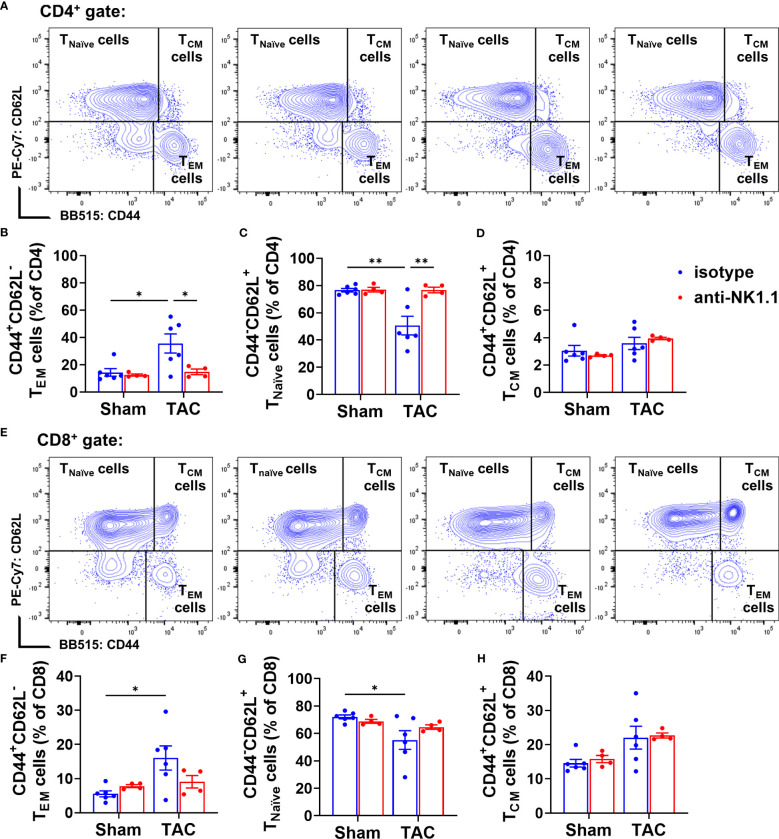
Effect of depletion of NK1.1^+^ cells on pulmonary CD4^+^ and CD8^+^ T cell activation. **(A-D)** Representative images of flow cytometry plots of pulmonary CD4^+^ T cells and the relative percentage of naïve (CD44^-^CD62L^+^CD4^+^), effector memory (CD44^+^CD62L^-^CD4^+^), and central memory (CD44^+^CD62L^+^CD4^+^) subsets. **(E–H)** Representative images of flow cytometry plots of and the percentage of pulmonary naïve (CD44^-^CD62L^+^CD8^+^), effector memory (CD44^+^CD62L^-^CD8^+^), and central memory cells (CD44^+^CD62L^+^CD8^+^) in CD8^+^ T cells. n=4-6. *p<0.05, **p<0.01.

Our previous studies showed that TAC causes cardiac and pulmonary T cell activation, while inhibition of T cell activation by CD28 KO, CD80/CD86 double KO, or induction of endogenous regulatory T cells was effective in attenuating TAC-induced HF and HF progression (5, 19). We further determined the effect of NK1.1^+^ cell depletion on activation of CD4^+^ and CD8^+^ T cells. We found that depletion of NK cells significantly attenuated TAC-induced activation of CD4^+^ T cells, as evidenced by an increased percentage of effector memory CD4^+^ T cells (CD44^+^CD62L^−^) and decreased percentage of naïve CD4^+^ T cells (CD44^-^CD62L^+^) in the CD4+ subset (**Figures 8A-D**). However, TAC-induced CD8^+^ T cell activation was not affected by the depletion of NK cells (**Figures 8E-H**). The percentages of central memory CD4^+^ and CD8^+^ T cells (CD44^+^CD62L^+^) were unaffected by TAC in both isotype-treated and anti-NK1.1 antibody-treated groups (**Figures 8D, H**).

### Depletion of NK1.1^+^ cells attenuated pulmonary infiltration and activation of macrophages and dendritic cells

**Figure 9:**
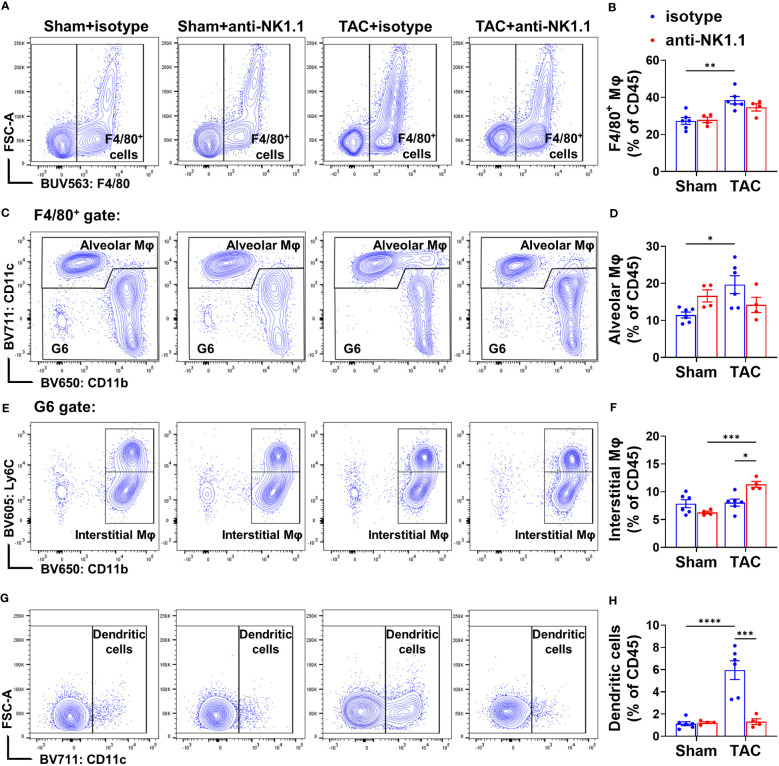
Effect of depletion of NK1.1^+^ cells on pulmonary macrophage and dendritic cell infiltration. **(A, B)** Representative images of flow cytometry plots of F4/80^+^ macrophages in the CD45^+^ population and relative percentages. n=4-6. **(C, D)** Representative images of flow cytometry plots of and relative percentage of F4/80^+^CD11c^+^ alveolar macrophages (AMφ) cells in the CD45^+^ subset. **(E, F)** Representative images of flow cytometry plots of and relative percentage of Ly6C^low^/CD11b^+^ interstitial Mφ (IMφ) in the CD45^+^ subset. **(G, H)** Relative percentage of CD11c^+^ dendritic cells in the CD45^+^ subset. n=4-6. *p<0.05, **p<0.01, ***p<0.001, ****p<0.0001.

Previous studies showed that NK1.1^+^ cells regulate the maturation and activation of macrophages and dendritic cells (DCs) in response to non-self or tumor antigens (42, 43). Since we previously observed that APCs regulate TAC-induced cardiac inflammation and HF (5), we further determined the accumulation and activation of pulmonary APCs by using a gating strategy illustrated in **Supplementary Figure S6**. We first examined the effect of depletion of NK1.1^+^ cells on the pulmonary macrophage population (F4/80^+^). TAC induced a significant increase in the percentage of pulmonary F4/80^+^ macrophages in the CD45^+^ population (**Figures 9A, B**). Depletion of NK1.1^+^ cells did not affect the percentage of total F4/80^+^ macrophages in the CD45^+^ population (**Figures 9A, B**). TAC with or without depletion of NK1.1^+^ cells did not change the percentages of IFN-γ^+^ macrophages, IL-1β^+^ macrophages, and TNF-α^+^ macrophages in the F4/80^+^ subset (**Supplementary Figure S7**). TAC caused a significant increase in the percentage of lung alveolar macrophages (AMϕ, F4/80^+^CD11c^+^ cells) (**Figures 9C, D**). Depletion of NK1.1^+^ cells did not affect the percentage of lung AMϕ or Ly6C^high^ monocyte-derived macrophages (MdMϕ, F4/80^+^CD11c^-^CD11b^+^Ly6C^high^) in the CD45^+^ population (**Figures 9C, D**, & **Supplementary Figure S8A**). The percentage of classical interstitial Ly6C^low^ macrophages (IMϕ) was not changed after TAC, but it was significantly increased in NK1.1^+^ cell-depleted TAC mice (**Figures 9E, F**). Moreover, depletion of NK1.1^+^ cells significantly attenuated the TAC-induced increase in the percentage of conventional CD11c^+^ DCs (Ly6G^-^F4/80^-^CD11c^+^) in mice (**Figures 9G, H**).

**Figure 10:**
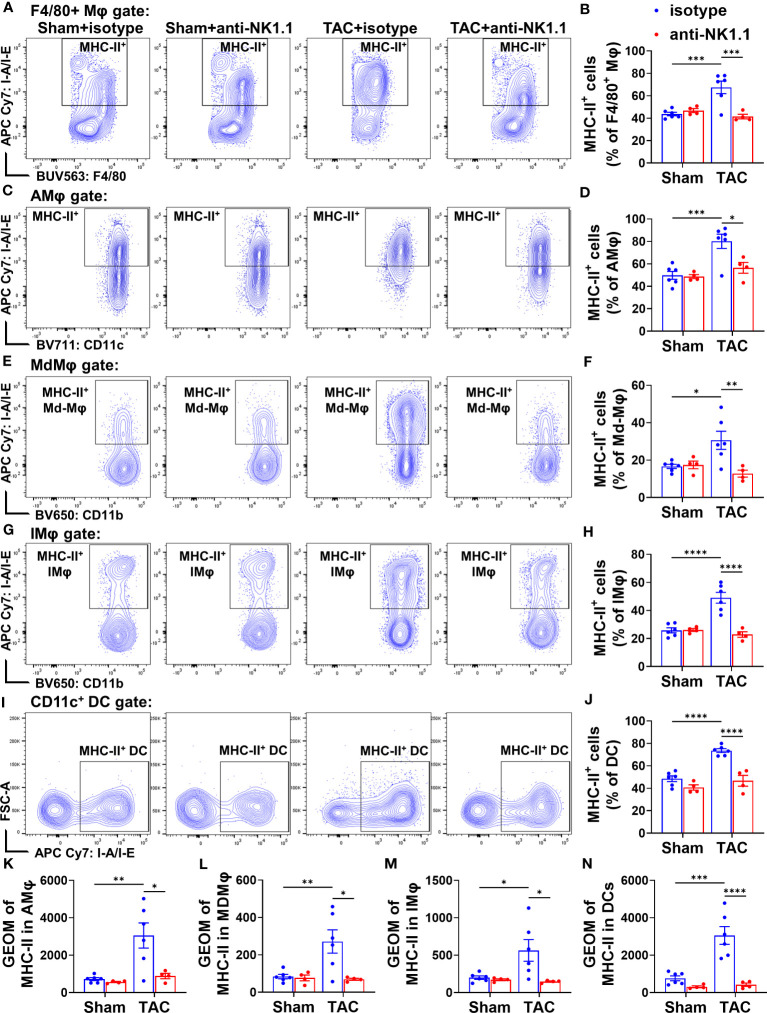
Effect of depletion of NK1.1^+^ cells on pulmonary macrophage and dendritic cell activation. **(A, B)** Representative images of flow cytometry plots and relative percentage of MHC-II^+^ cells in the total F4/80^+^ Mφ subset. n=4-6. **(C, D)** Representative images of flow cytometry plots and relative percentage of MHC-II^+^ cells in the AMφ subset. n=4-6. **(E, F)** Representative images of flow cytometry plots of and relative percentage of MHC-II^+^ cells in the MdMφ population. **(G, H)** Representative images of flow cytometry plots of and relative percentage of MHC-II^+^ cells in the IMφ population. **(I, J)** Relative percentage of MHC-II^+^ cells in the dendritic cell subset. n=4-6. **(K-N)** Geometric mean of fluorescent intensity of MHC-II in the AMφ, MDMφ, IMφ, and DC population. *p<0.05, **p<0.01, ***p<0.001, ****p<0.0001. GEO mean, geometric mean.

Since MHC-II expression in macrophages or DCs often reflects their activation status, MHC-II expressions in the above cells were determined. TAC resulted in significant increases of MHC-II expression in the overall F4/80^+^ macrophages, F4/80^+^CD11c^+^CD11b^-^ AMϕ, Ly6C^high^MdMϕ, and the classical Ly6C^low^ IMϕ (**Figures 10A-H**). Depletion of NK1.1^+^ cells significantly attenuated TAC-induced increases of MHC-II expression in the total F4/80^+^ macrophage population, and in their corresponding AMϕ, Ly6C^high^ MdMϕ, and Ly6C^low^ classical IMϕ subsets (**Figures 10A-H**). Moreover, TAC resulted in a significant increase of MHC-II expression in DCs (**Figures 10I, J**), while depletion of NK1.1^+^ cells significantly attenuated the TAC-induced MHC-II expression in DCs (**Figures 10I, J**). Furthermore, TAC resulted in significant increases in the GEO mean of MHC-II in the corresponding AMϕ, MdMϕ, IMϕ, and DC subsets, which were attenuated by depletion of NK1.1^+^ cells (**Figures 10K-N**). These data indicate that depletion of NK1.1^+^ cells attenuated the TAC-induced infiltration and activation of pulmonary macrophages and DCs.

Neither TAC nor depletion of NK1.1^+^ cells significantly affected the accumulation of pulmonary B cells (CD19^+^MHC-II^+^) or neutrophils (Ly6G^+^CD11b^+^) (**Supplementary Figures S8B, C**).

## Discussion

**Figure 11:**
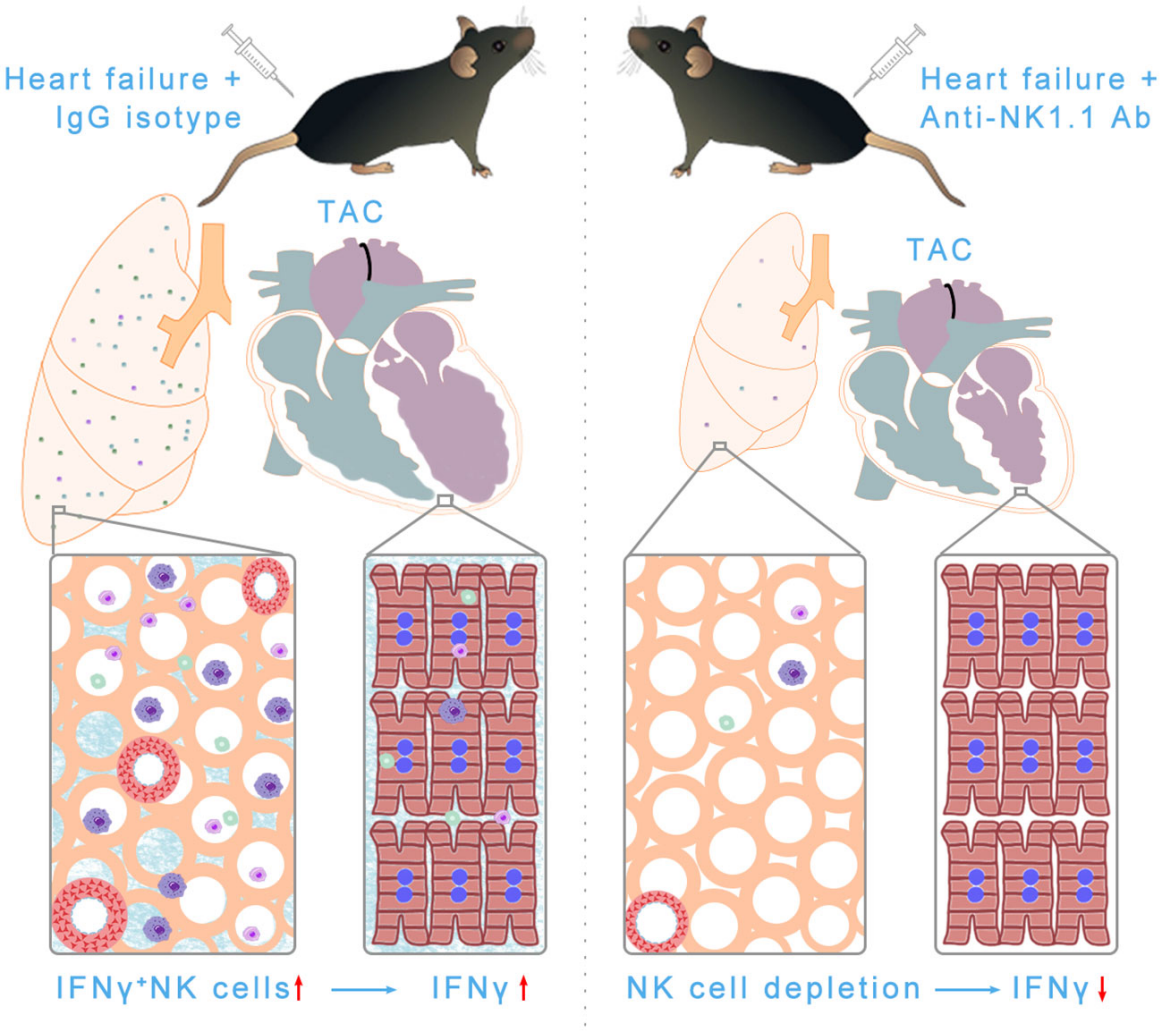
Schematic showing NK and NKT-derived IFN-γ was significantly increased in pulmonary tissues after HF. Depletion of NK/NKT cells using anti-NK1.1 blocking antibodies significantly attenuated systolic overload-induced left ventricular inflammation, fibrosis, and dysfunction, as well as pulmonary leukocyte infiltration, fibrosis, vessel remodeling, and right ventricular hypertrophy. In summary, NK and NKT cells regulate systolic overload-induced HF development and progression by modulating cardiopulmonary inflammatory response.

The present study has several major new findings. First, we demonstrated for the first time that pulmonary IFN-γ^+^ NK1.1^+^NK cells and IFN-γ^+^ NK1.1^+^NKT cells are significantly increased after HF development. After HF, NK1.1^+^ cells account for ~50% of pulmonary IFN-γ^+^ cells. Second, we found that systemic depletion of NK1.1^+^ cells significantly attenuated TAC-induced LV dysfunction, leukocyte infiltration, and fibrosis in immunocompetent C57BL/6J mice. Third, systemic depletion of NK1.1^+^ cells significantly attenuated TAC-induced pulmonary inflammation, fibrosis, and vessel remodeling, as well as RV remodeling. Furthermore, we found that depletion of NK1.1^+^ cells attenuated TAC-induced accumulation and activation of pulmonary macrophages, dendritic cells, CD4^+^ T cells, and CD8^+^ T cells. Together, these findings indicate that NK1.1^+^ cells exert an important role in promoting systolic overload-induced HF development and the progression from LV failure to lung remodeling and RV hypertrophy in immunocompetent mice (**Figure 11**).

Immune cells, particularly lymphocytes, have recently emerged as a focus for their roles in regulating various cardiopulmonary inflammation and HF development. Specifically, cardiac inflammation promotes cardiomyocyte injury, fibrosis, LV dysfunction, and lung remodeling. Pulmonary inflammation directly promotes lung injury, fibrosis, vessel remodeling, and the consequent RV failure (3–6). Thus, one study showed that CD4^+^ T cell KO attenuated TAC-induced HF and the transition from LV hypertrophy to LV dysfunction (20). Another study demonstrated that Th1 effector T cells contribute to TAC-induced cardiac hypertrophy, fibrosis, and dysfunction (18). We demonstrated that activated T cells were increased in LV and lung tissues in HF mice (5), while depletion of CD11c+ antigen-presenting cells or inhibition of the crosstalk between T cells and APCs by CD28 knockout or CD86/CD80 (B7) double knockout significantly attenuated TAC-induced LV hypertrophy and failure (16, 19). In addition, inhibition of inflammation by induction of endogenous regulatory T cells effectively attenuated the transition from LV failure to lung remodeling and RV hypertrophy in mice with existing LV failure (5). Recently, we further demonstrated that IL12α KO significantly attenuated TAC-induced LV hypertrophy and failure, pulmonary T cell activation, inflammation, fibrosis, and vessel remodeling (44). Moreover, studies from other groups also demonstrated that LV NK cells, residential macrophages, and monocyte-derived macrophages were significantly increased in mice after TAC (31, 45). These studies have demonstrated the important roles of immune cells, particularly lymphocytes, in regulating HF development and progression through modulating LV and/or pulmonary inflammation. The present study further provides direct evidence that NK1.1^+^ lymphocytes contribute to TAC-induced cardiac inflammation and dysfunction, as well as the consequent pulmonary inflammation, vessel remodeling, and RV hypertrophy in immune-competent mice.

The important findings of this study include the increased pulmonary IFN-γ^+^ NK1.1^+^NK and NK1.1^+^NKT cells after HF, suggesting that NK1.1^+^ cells exert an important role in HF-induced lung inflammation and tissue IFN-γ^+^ bioavailability. Indeed, the depletion of NK1.1^+^ cells significantly attenuated TAC-induced pulmonary inflammation, fibrosis, and vessel remodeling. Since LV failure causes pulmonary inflammation and remodeling (3, 5) and since lung inflammation could further promote pulmonary fibrosis and vessel remodeling, without affecting LV hypertrophy and/or failure (6), the significantly reduced lung inflammation, fibrosis, and vessel remodeling after depletion of NK1.1^+^ cells are likely a collective effect of reducing LV dysfunction and directly attenuating lung inflammation. However, as TAC often causes more pulmonary inflammation than cardiac inflammation after the development of severe HF (3, 5), depletion of NK1.1^+^ cells likely exerts a more important role in reducing pulmonary inflammation and vessel remodeling than its effect on LV tissue. The finding that depletion of NK1.1^+^ cells had no significant effect on TAC-induced LV hypertrophy supports the notion that NK1.1^+^ cells might exert a relatively mild effect on TAC-induced LV inflammation and remodeling. The findings that TAC resulted in increased infiltration and activation of pulmonary residential macrophages, Ly6C^+^ monocyte-derived macrophages, dendritic cells, and T cells are consistent with our previous reports (4, 5, 43). The reduced RV leukocyte infiltration, fibrosis, and hypertrophy in mice after depletion of NK1.1^+^ cells are likely a collective outcome of reduced pulmonary remodeling and their direct anti-inflammatory effect on RV tissues.

While the findings that depletion of NK1.1^+^ cells significantly attenuated rather than promoted TAC-induced pulmonary inflammation, fibrosis, vessel remodeling, and RV hypertrophy are striking, these findings are not totally unanticipated. First, previous studies have clearly demonstrated that idiopathic PH is associated with pulmonary NK deficiency or reduced lung residential NK1.1^+^NK cells (25), and idiopathic pulmonary fibrosis-induced PH in patients and mice are associated with NKT cell deficiency without affecting NK cell infiltration (24). While the degree of pulmonary NK1.1^+^ cell infiltrations in lung tissues from patients with HF is unknown, our study showed that both pulmonary NK1.1^+^ cells are increased after TAC-induced HF in wild type C57BL/6J mice, indicating that pulmonary NK1.1^+^ cells are increased after HF as compared to the reduced NK and NKT cells in idiopathic PH or fibrosis-induced PH. Second, previous studies clearly demonstrated that IFN-γ plays an important role in promoting angiotensin-II or TAC-induced cardiac inflammation, fibrosis, and HF (18, 32). NK1.1^+^ cells contribute to IFN-γ^+^ production, and our study further showed that pulmonary IFN-γ^+^ NK cells and IFN-γ^+^ NKT cells were increased after HF. Thus, depletion of NK1.1^+^ cells could protect against HF-induced lung inflammation and injury by reducing IFN-γ production by NK1.1^+^NK and NK1.1^+^NKT cells. Third, the NK cell-deficient mouse strains are immune-impaired or NK dysfunctional mouse strains, while the mice used in the present study were immune competent adult mice. In addition, the global gene deficiency of NKp46 and Nfil3 might cause potential unknown adaptive changes to alter their effect on PH development in these strains. Fourth, the different mouse strains and experimental models may also contribute to the different phenotypes. Furthermore, the mechanism of idiopathic PH and remodeling might be different from HF-induced PH and remodeling as previous studies clearly demonstrated that the commonly used effective therapies for idiopathic PH (such as PDE5 inhibitors or endothelin receptor antagonists) were not effective in treating HF-induced PH (46), suggesting the underlying mechanisms of these pulmonary vessel remodeling and PH are different.

### Study limitations

The present study has several limitations. First, depletion of NK1.1^+^ cells attenuated TAC-induced LV dysfunction, cardiopulmonary inflammation and fibrosis, pro-inflammatory cytokine production, and the activation of T cells and antigen-presenting cells. Since all the above factors/changes could regulate HF development and/or progression, the current study could not differentiate the relative contribution of each of these factors. Nevertheless, the findings from the present study still provide important mechanistic insights for systolic overload-induced HF development and progression. Second, we only studied male mice in the present study. In the context that sex is an important biological factor that affects TAC-induced HF development, there is a possibility that female and male mice may have different degrees of cardiopulmonary inflammation and remodeling after depletion of NK1.1^+^ cells. However, as cardiopulmonary inflammation, LV hypertrophy and dysfunction, pulmonary fibrosis, and remodeling are commonly observed in both male and female mice after TAC, and since NK and NKT cells exert similar roles in both male and female mice in various disease conditions, NK1.1^+^ cell depletion is anticipated to exert similar impacts on TAC-induced HF progression in both sexes. Third, like other studies in the field, our study could not differentiate the relative effect of NK1.1^+^NK cells and NK1.1^+^NKT cells on HF development. However, the observed effects are probably mainly due to the NK1.1^+^NK cells, as NK1.1^+^NKT cells are relatively rare compared with NK1.1^+^NK cells. Nevertheless, the observed phenotypes in the current study are likely the collective effects of both NK1.1^+^NK and NK1.1^+^NKT cells. Fourth, HF-induced lung vessel remodeling and consequent RV hypertrophy are often associated with increased pulmonary artery pressure. Unfortunately, to obtain high-quality pulmonary samples for immunological experiments, pulmonary artery pressure was not determined in our study. Nevertheless, the lung vessel remodeling and the degree of RV hypertrophy clearly indicate that pulmonary pressure was affected in mice after TAC. Moreover, although it is well-known that NK cell trafficking and homing are regulated by integrins, selectins, and chemokine receptors (such as CCR5, CXCR6, and CXCR3, etc.) (47), we did not determine the factors that modulate pulmonary NK1.1 cell homing. Finally, as in most experimental studies, the present study was performed in young and healthy immunocompetent C57BL/6J mice under SPF conditions (relative sterile conditions). Since HF often occurs in older patients exposed to chronic respiratory pathogens or pollution etc., the findings from these healthy young experimental animals, without exposure to pathogens or respiratory burdens, might not fully reflect the clinical conditions. Thus, the interpretation of the present findings should be cautious.

### Conclusions

We demonstrated that pulmonary NK1.1^+^ cells and their IFN-γ^+^ cell subsets are increased after HF in immunocompetent mice, and depletion of NK1.1^+^ cells significantly attenuated systolic overload-induced cardiopulmonary inflammation, LV dysfunction, lung vessel remodeling, and RV hypertrophy. These findings indicate that NK1.1^+^ cells promote systolic overload-induced HF development and HF progression in immunocompetent mice likely through a pathway related to IFN-γ production.

## Data availability statement

The raw data supporting the conclusions of this article will be made available by the authors, without undue reservation.

## Ethics statement

The animal study was reviewed and approved by Institutional Animal Care and Use Committee at the University of Mississippi Medical Center.

## Author contributions

XH, RX, XL, LP, UB, and YC contributed to data collection and analysis. XH and YC contributed to experimental design and manuscript preparation. J-XC, HZ, LP, UB, MH, and YC edited the manuscript. All authors contributed to the article and approved the submitted version.

## Glossary

**Table:**

AMϕ	alveolar macrophages
APCs	antigen-presenting cells
CSA	cross-sectional area
DCs	dendritic cells
EF	ejection fraction
FS	fractional shortening
GEO mean	geometric mean
GZMA	granzyme A
HF	heart failure
IFN-γ	interferon gamma
IL-1β	interleukin-1beta
IL6	interleukin-6
IMϕ	interstitial macrophages
KO	knockout
LV	left ventricular
LVESD	LV end-systolic diameter
LVEDD	LV end-diastolic diameter
LVESV	LV end-systolic volume
LVEDV	LV end-diastolic volume
MdMϕ	monocyte-derived macrophages
MHC-II	major histocompatibility complex class II
NK	natural killer
NKT	natural killer T
PBS	phosphate-buffered saline
PRF	perforin
PH	pulmonary hypertension
RV	right ventricular
TAC	transverse aortic constriction
TNF-α	tumor necrosis factor-alpha
WGA	wheat germ agglutinin
