# Supplementary material for: Inhibition of NK1.1 signaling attenuates pressure overload-induced heart failure, and consequent pulmonary inflammation and remodeling
Source: Front Immunol. 2023 Jul 24;14:1215855. doi: 10.3389/fimmu.2023.1215855 (PMC10405176; doi:10.3389/fimmu.2023.1215855)
Supplement: Supplementary file 1 [file DataSheet_1.docx]

Supplementary Material

**Inhibition of NK1.1 signaling attenuates pressure overload-induced heart failure, and consequent pulmonary inflammation and remodeling**

**Xiaochen He^1†^, Rui Xu^1†^, Lihong Pan^1^, Umesh Bhattarai^1^, Xiaoguang Liu^1,2^, Heng Zeng^3^,**

**Jian-Xiong Chen^3^, Michael E. Hall^1,4^, Yingjie Chen^1^**

^1^Department of Physiology and Biophysics, University of Mississippi Medical Center, School of Medicine, Jackson, MS, 39216, USA

^2^ College of Sports and Health, Guangzhou Sport University, Guangzhou 510500, China

^3^ Department of Pharmacology and Toxicology, University of Mississippi Medical Center, School of Medicine, Jackson, MS, 39216, USA

^4^ Department of Medicine, University of Mississippi Medical Center, School of Medicine, Jackson, MS, 39216, USA

†These authors contributed equally to this work and share first authorship

**Address for Correspondence:**

Yingjie Chen, Ph.D.

Department of Physiology and Biophysics,

University of Mississippi Medical Center,

2500 North State Street,

Jackson, MS, 39216

Office: 601-815-3986

Email: [ychen2@umc.edu](mailto:ychen2@umc.edu)

**Supplementary Figures:
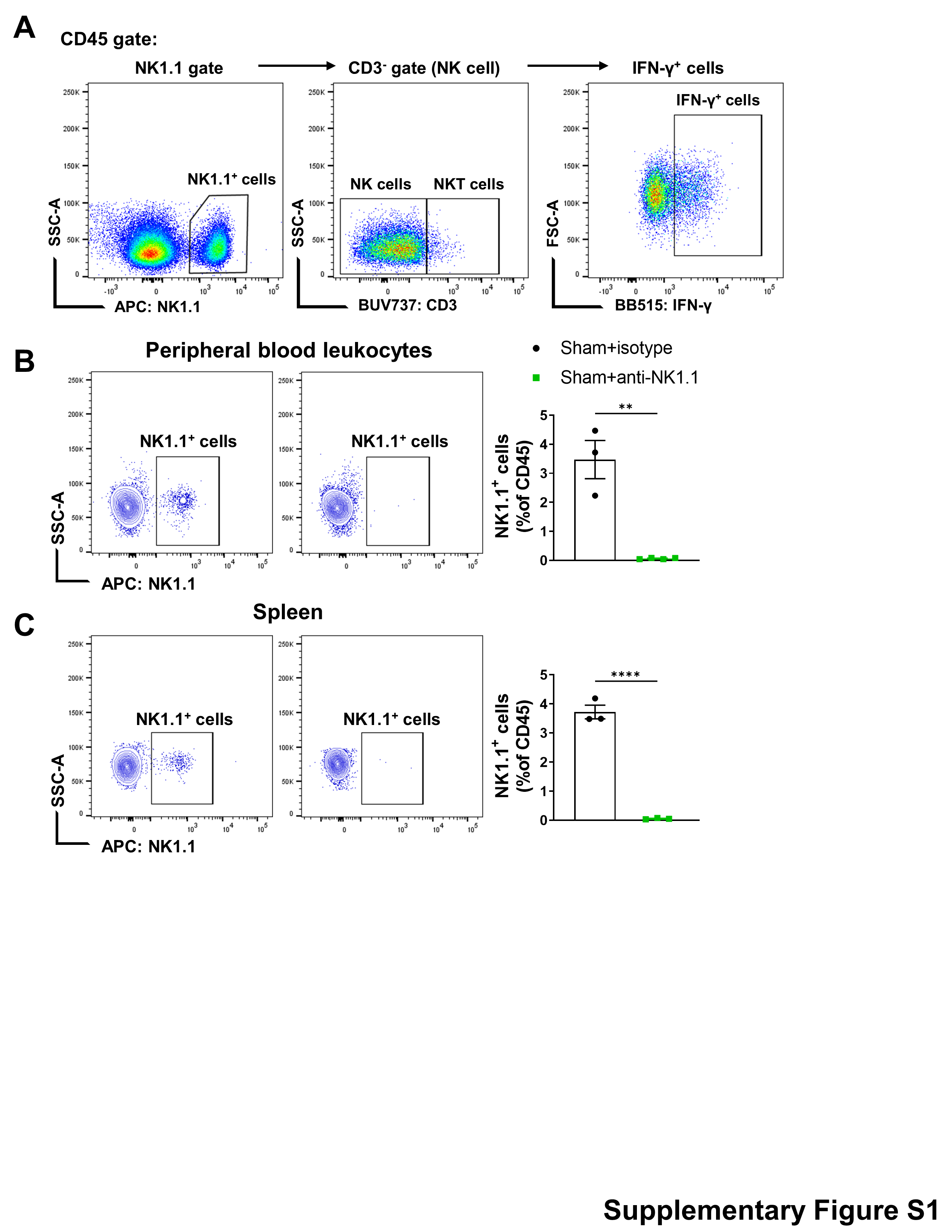
**

**Anti-NK1.1**

**Isotype**

**Isotype**

**Anti-NK1.1**

**Supplementary Figure S1. A,** Gating strategy for flow cytometry analysis of NK1.1^+^ cells, NK cells NKT cells, and IFN-γ^+^ cells in peripheral blood immune cells and spleen. **B&C,** Representative images of flow cytometry plots of and relative percentage of NK1.1^+^ cells in the CD45^+^ subset of peripheral blood leukocytes and spleen, respectively. n=3-4. **p<0.01, ****p<0.0001.


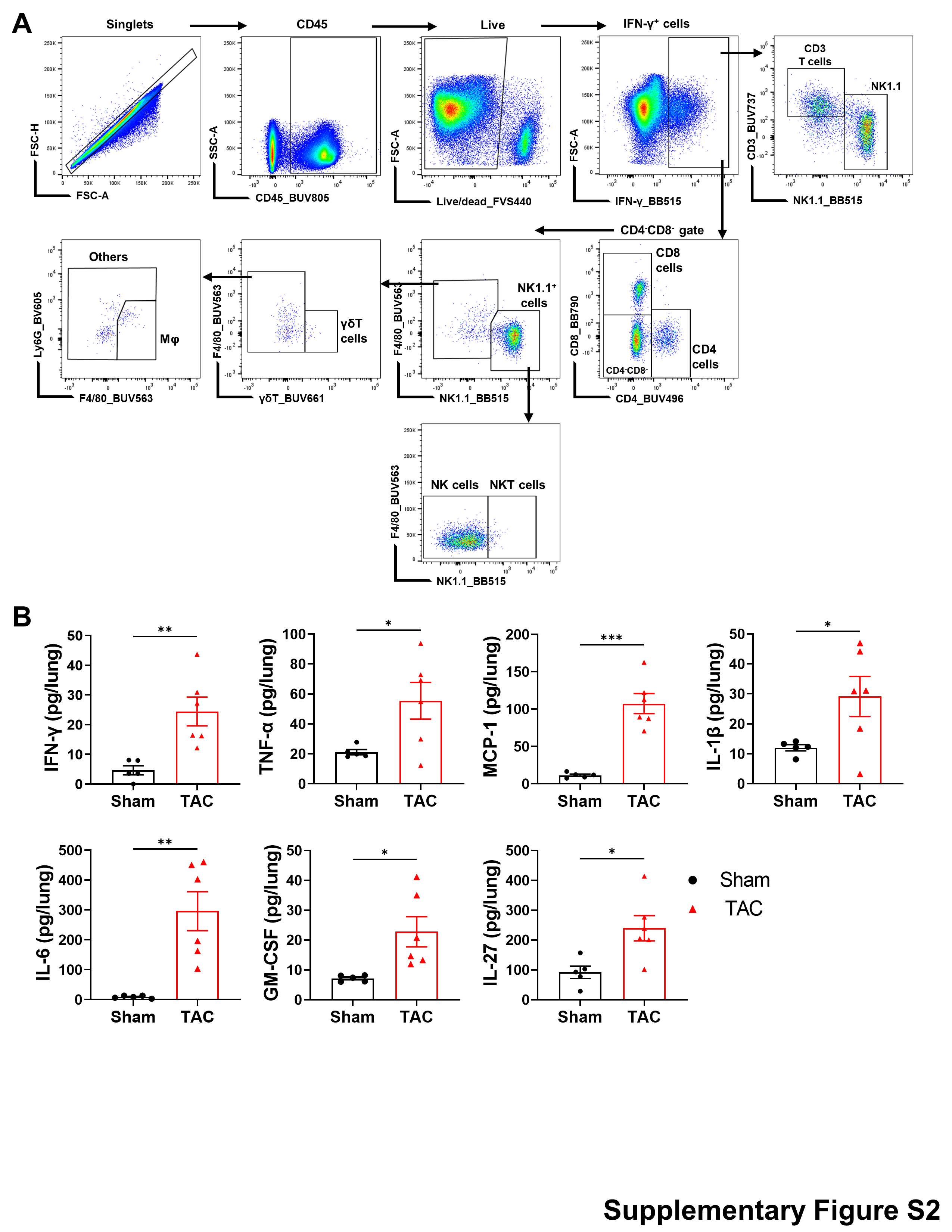


**Supplementary Figure S2. A,** Gating strategy for flow cytometry analysis of pulmonary CD3, CD4, CD8, γδT, NK1.1^+^NK, NK1.1^+^NKT, macrophage (Mφ), and other cells in the IFN-γ^+^ subset. **B,** Cytokine content in the lung of sham or TAC mice measured by bead-based cytokine assay. n=4-6. *p<0.05, **p<0.01, ***p<0.001.


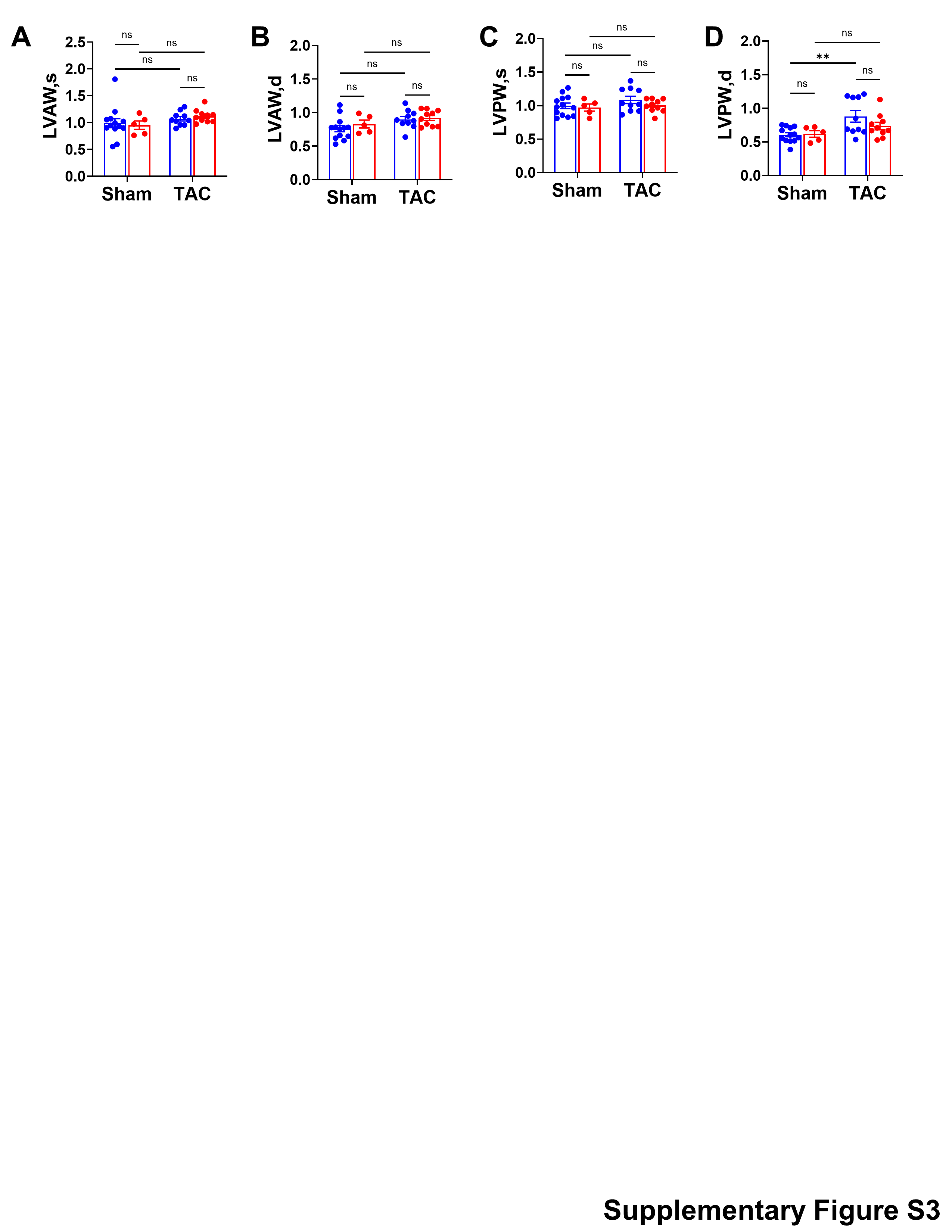


**Supplementary Figure S3. A-D,** Echocardiographic measurements of LV wall thickness at end-systole and end-diastole. n=5-13. AW, anterior wall; PW, posterior wall; ns, not significant; **p<0.01.


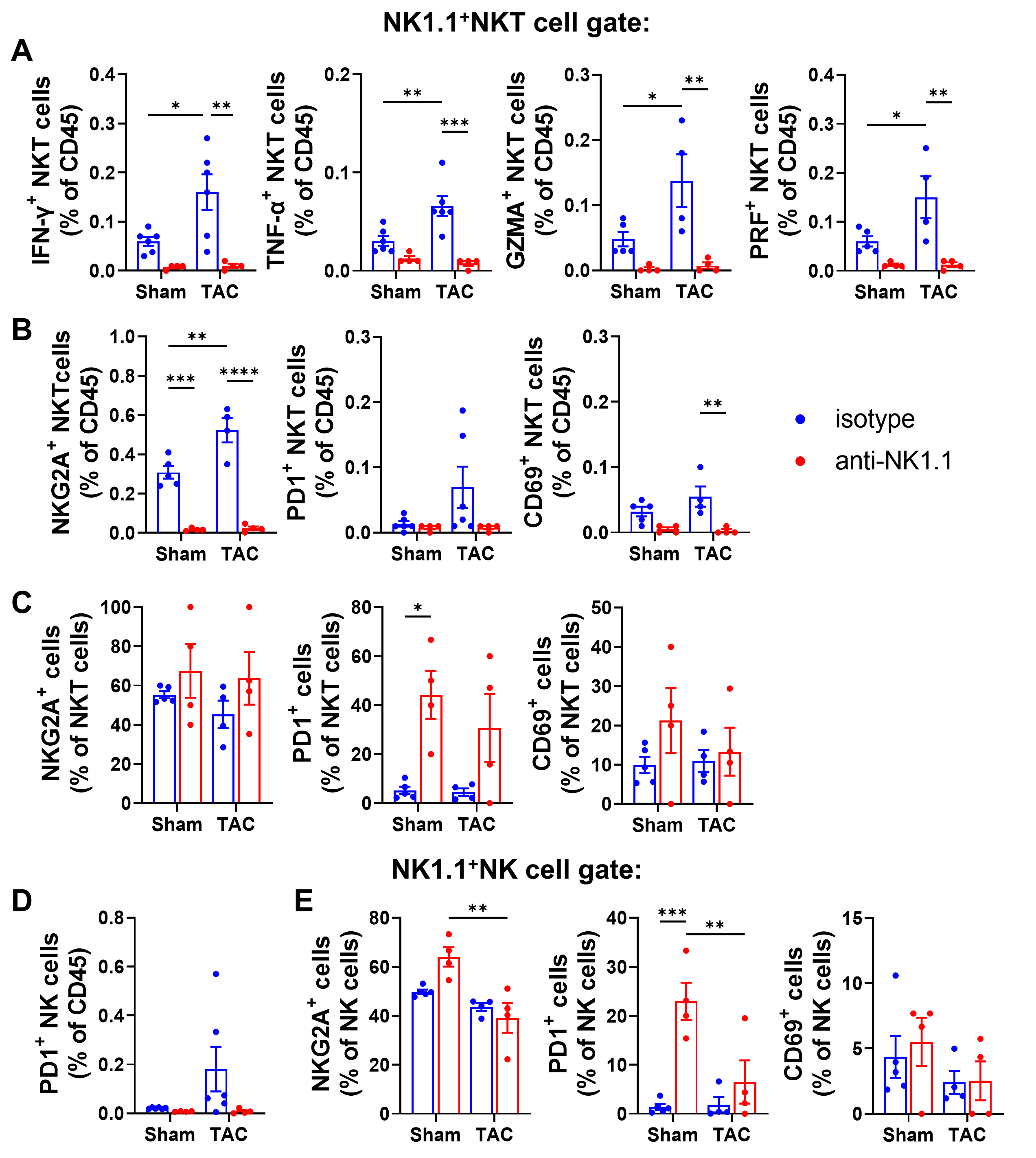


**Supplementary Figure S4. A,** Percentage of pulmonary IFN-γ^+^, TNF-α^+^, granzyme A (GZMA)^+^, perforin-1 (PRF)^+^ NK1.1^+^NKT cells in the CD45^+^ subset. **B,** Percentage of NKG2A^+^, PD1^+^, CD69^+^ NK1.1^+^NKT cells in the CD45^+^ subset. **C,** Percentage of NKG2A^+^, PD1^+^, CD69^+^ cells in the NK1.1^+^NKT cell subset. **D,** Percentage of PD1^+^ NK1.1^+^NK cells in the CD45^+^ subset of lung tissue. **E,** Percentage of NKG2A^+^, PD1^+^, CD69^+^ cells in NK1.1^+^NK subset. n=4-6. *p<0.05, **p<0.01, ***p<0.001, ****p<0.0001. GZMA: granzyme A; PRF: perforin.


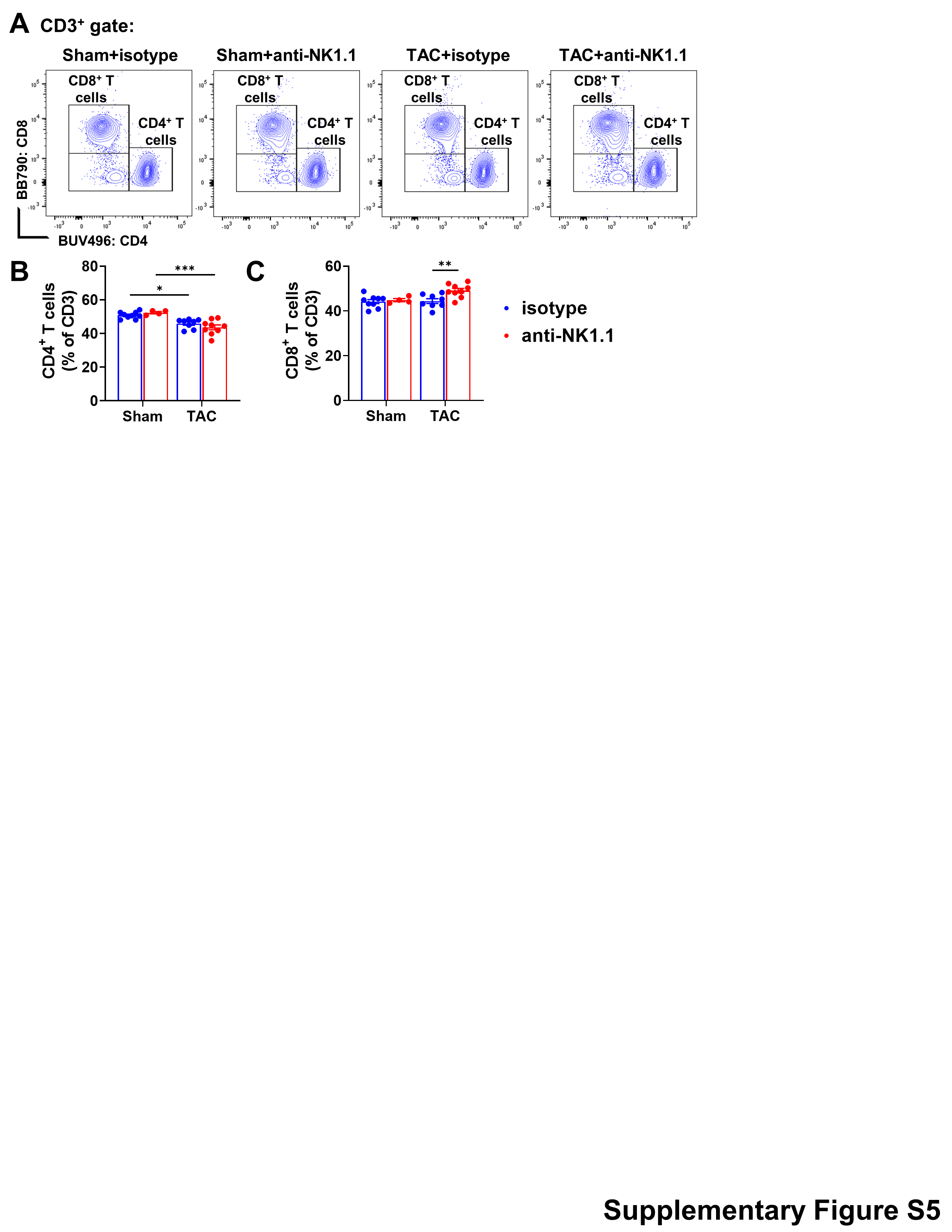


**Supplementary Figure S5. A-C,** Representative images of flow cytometry plots of and relative percentages of pulmonary CD4^+^ and CD8^+^ T cells in the CD3^+^ T cell subset. n=4-9. *p<0.05, **p<0.01, ***p<0.001.


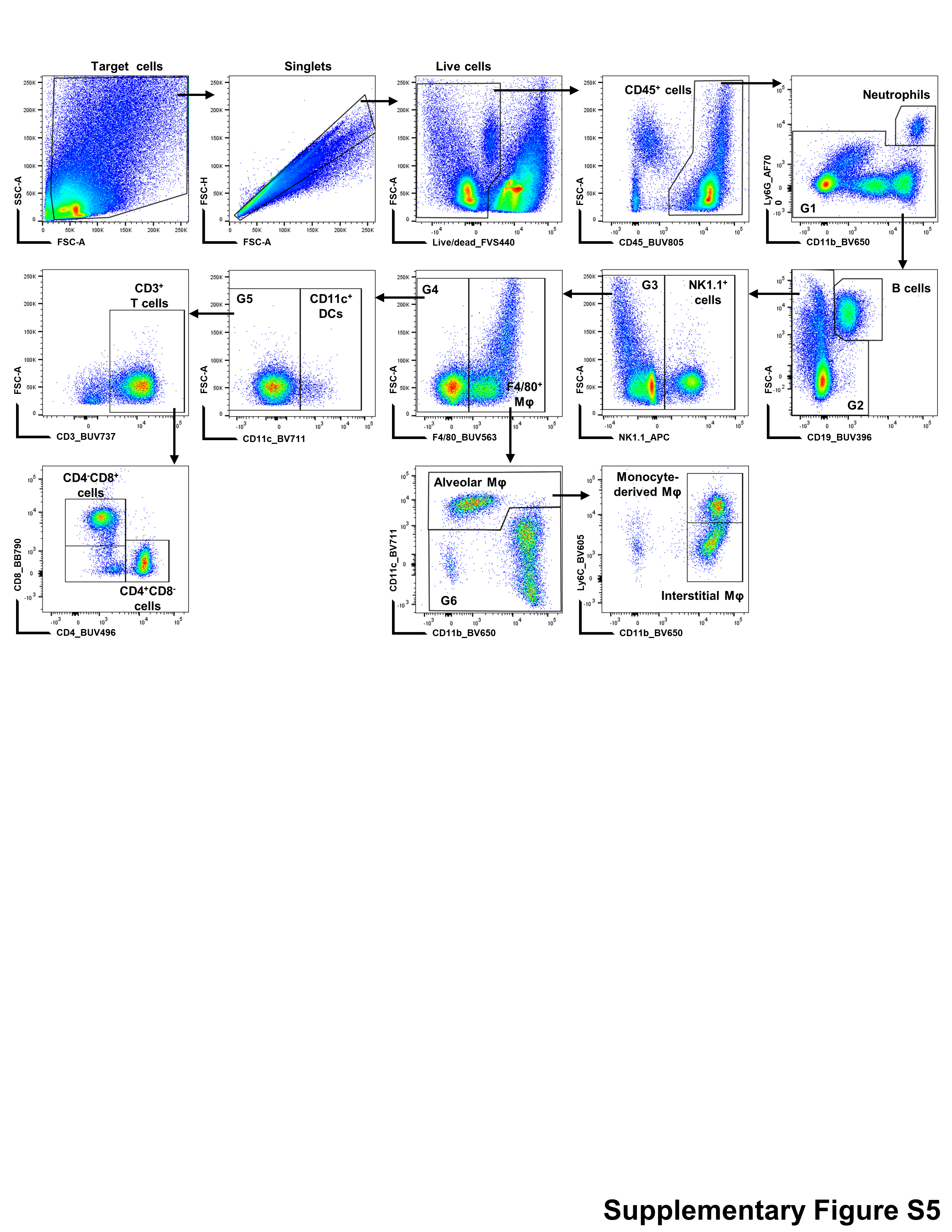


**Supplementary Figure S6.** Gating strategy for flow cytometry analysis of pulmonary antigen-presenting cells (APCs), neutrophils, NK1.1^+^NK cells, and T cells in mice.


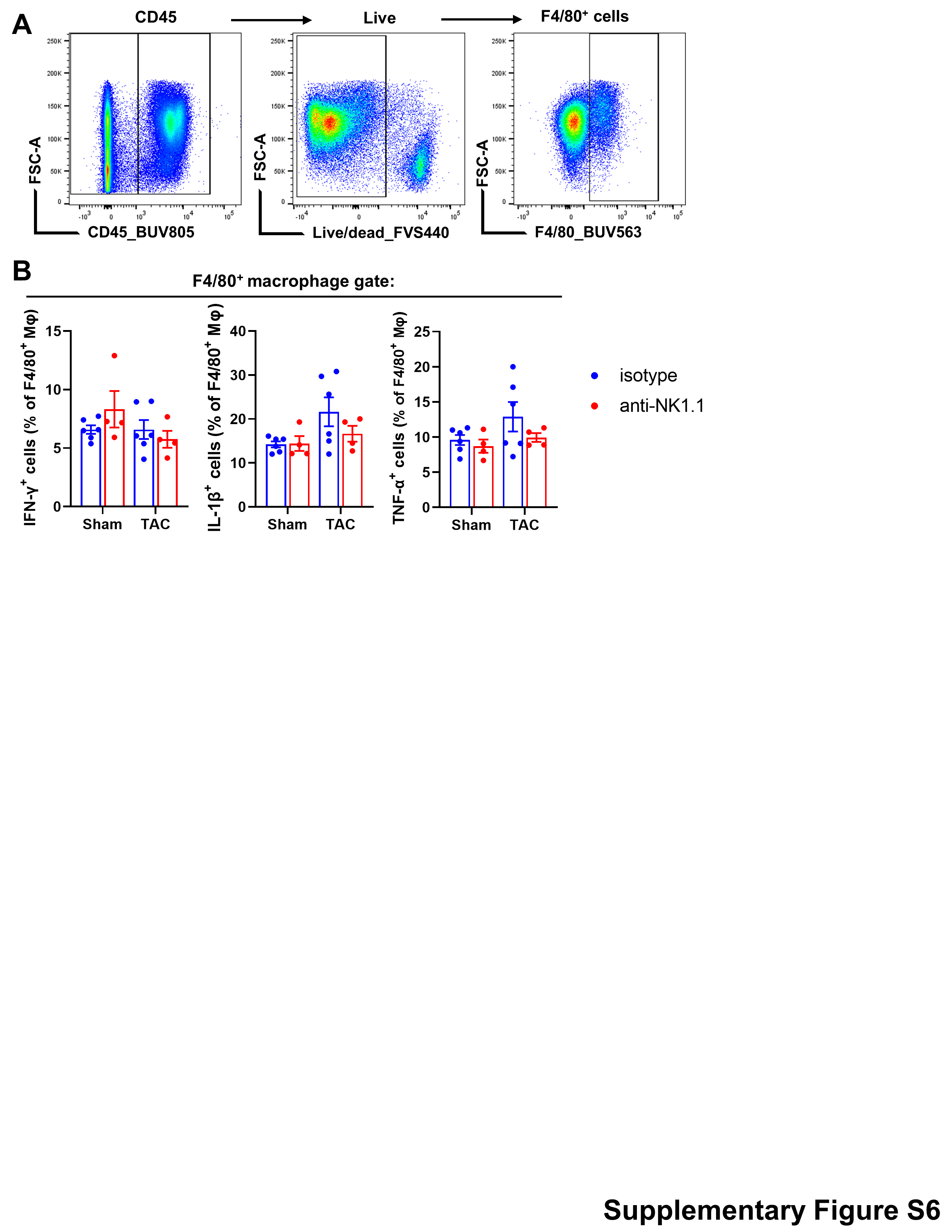


**Supplementary Figure S7. A&B,** Gating strategy for flow cytometry analysis of pulmonary F4/80^+^ macrophages for cytokine production assay and relative percentage of IFN-γ^+^, IL-1β^+^, and TNF-α^+^ cells in the F4/80^+^ macrophages. n=4-6. *p<0.05.


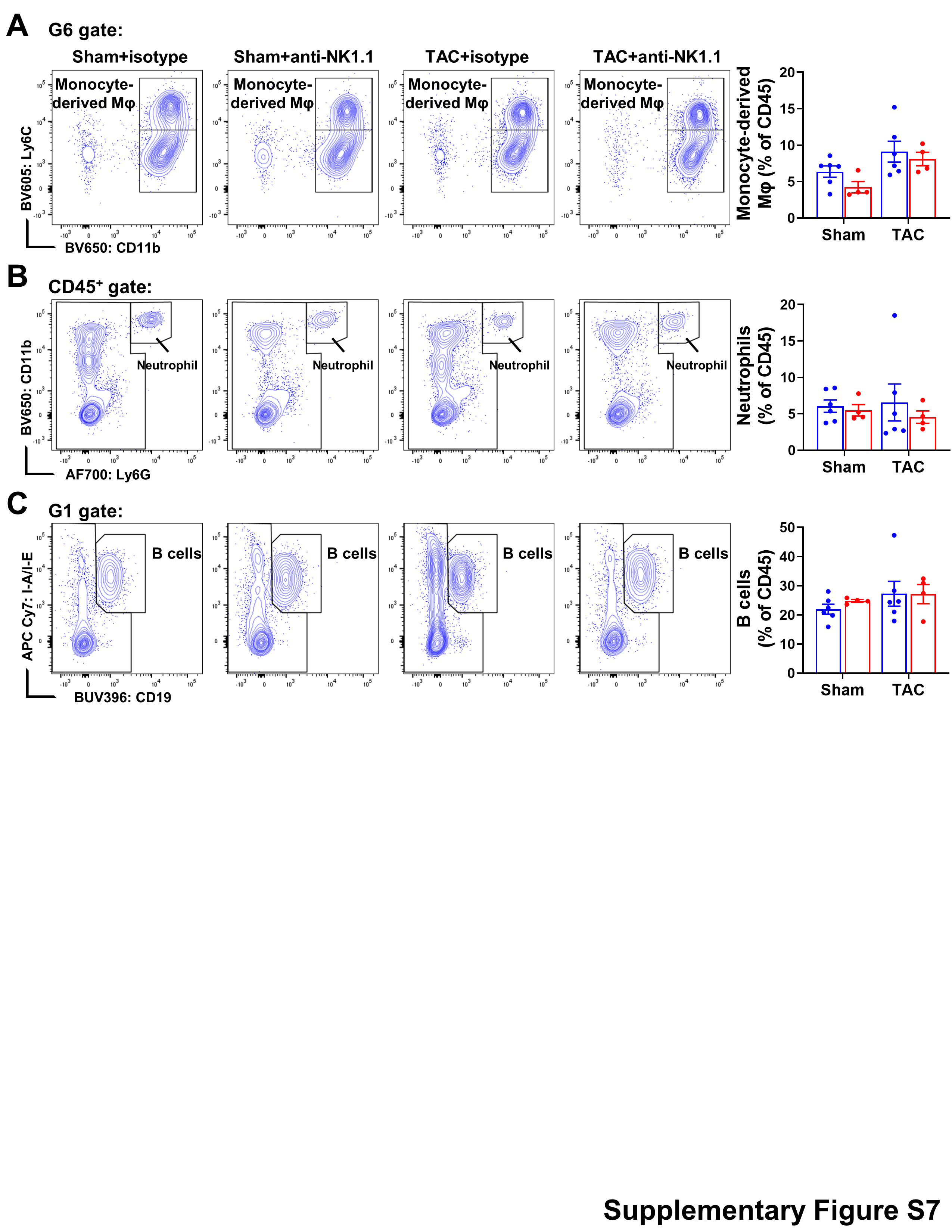


**Supplementary Figure S8. A,** Representative images of flow cytometry plots of and the percentage of pulmonary Ly6C^high^/CD11b^+^ monocyte-derived Mφ (MdMφ) in the CD45^+^ subset. **B,** Representative images of flow cytometry plots of and relative percentage of Ly6G^+^/CD11b^+^ neutrophils in the CD45^+^ subset. **C,** Representative images of flow cytometry plots of and relative percentage of CD19^+^/MHC-II^+^ B cells in the CD45^+^ subset. n=4-6.

| **Supplementary Table S1. Primary antibodies used for flow cytometry analysis** | | | | |
| --- | --- | --- | --- | --- |
| **Primary Antibody** | **Conjugate** | **Clone** | **Vender** | **Catalog #** |
| **CD3e** | BUV737 | 145-2C11 | BD Biosciences | 612771 |
| **CD4** | BUV496 | GK1.5 | BD Biosciences | 612952 |
| **CD8α** | BB790 | 53-6.7 | BD Biosciences | 624296* |
| **CD11b** | BV650 | M1/70 | Biolegend | 101259 |
| **CD11c** | BV711 | N418 | Biolegend | 117349 |
| **CD16/32** | - | 93 | Biolegend | 101302 |
| **CD19** | BUV395 | 1D3 | BD Biosciences | 563557 |
| **CD44** | FITC | IM7 | BD Biosciences | 553133 |
| **CD45** | BUV805 | 30-F11 | BD Biosciences | 748370 |
| **CD62L** | PE-Cy7 | MEL-14 | Biolegend | 104418 |
| **CD69** | AF700 | H1.2F3 | Biolegend | 104539 |
| **CD279 (PD1)** | BV480 | J43 | BD Biosciences | 746784 |
| **F4/80** | BUV563 | T45-2342 | BD Biosciences | 749284 |
| **Granzyme A** | PE | 3G8.5 | Biolegend | 149704 |
| **γδ T-Cell Receptor** | BUV661 | GL3 | BD Biosciences | 750410 |
| **I-A/I-E (MHC-II)** | APC-Cy7 | M5/114.15.2 | Biolegend | 107628 |
| **IFN-γ** | FITC | XMG1.2 | Biolegend | 505806 |
| **IL-1β** | PE-Cy7 | NJTEN3 | eBioscience | 25-7114-82 |
| **Ly6C** | BV605 | AL-21 | BD Biosciences | 563011 |
| **Ly6G** | AF700 | 1A8 | Biolegend | 127622 |
| **Ly6G** | BV605 | 1A8 | BD Biosciences | 563005 |
| **NK1.1** | APC | PK136 | eBioscience | 17-5941-82 |
| **Perforin** | PE-Dazzle594 | S16009A | Biolegend | 154316 |
| **TNF-α** | BV650 | MP6-XT22 | BD Biosciences | 563943 |

*Custom order

| **Parameters** | **Sham**  **+isotype** | **Sham**  **+anti-NK1.1** | **TAC**  **+isotype** | **TAC**  **+anti-NK1.1** |
| --- | --- | --- | --- | --- |
| **Number** | n=13 | n=5 | n=10 | n=10 |
| **Heart Rate (bpm)** | 491±11 | 519±14 | 482±6 | 489±13 ^ns^ |
| **LVESV (µL)** | 19.63±1.80 | 16.52±1.83 | 64.57±8.88* | 46.28±8.40 |
| **LVEDV (µL)** | 59.76±1.66 | 58.27±3.50 | 98.31±8.58* | 82.95±7.99 |
| **SV (µL)** | 40.13±1.29 | 41.75±1.93 | 33.74±3.60 | 36.67±2.55 ^ns^ |
| **CO (mL/min)** | 19.75±0.82 | 21.70±1.17 | 16.31±1.81 | 17.77±1.09 ^ns^ |
| **LV Mass (mg)** | 85.51±4.58 | 90.59±6.16 | 176.59±21.53* | 140.52±14.56 |
| **LVAW;s (mm)** | 0.99±0.08 | 0.95±0.08 | 1.07±0.04 | 1.12±0.04 ^ns^ |
| **LVAW;d (mm)** | 0.76±0.05 | 0.83±0.06 | 0.90±0.04 | 0.92±0.04 ^ns^ |
| **LVPW;s (mm)** | 1.00±0.04 | 0.97±0.05 | 1.09±0.06 | 1.00±0.03 ^ns^ |
| **LVPW;d (mm)** | 0.61±0.03 | 0.62±0.05 | 0.88±0.09* | 0.74±0.06 |

**Supplementary Table S2.** **Echocardiographic data in experimental groups.**

Data are shown as mean ± SEM. LVESV, left ventricular end-systolic volume; LVEDV, left ventricular end-diastolic volume; SV, stroke volume; CO, cardiac output; LVAW, left ventricular anterior wall; LVPW, left ventricular posterior wall. *p<0.05 vs. corresponding sham mice, ns, not significant for all groups.

| **Parameters** | **Sham**  **+isotype** | **Sham**  **+anti-NK1.1** | **TAC**  **+isotype** | **TAC**  **+anti-NK1.1** |
| --- | --- | --- | --- | --- |
| **Number** | n=22 | n=5 | n=23-26 | n=22-23 |
| **BW (g)** | 28.4 ±0.8 | 25.8±0.7 | 29.4±0.7 | 28.8±0.5 ^ns^ |
| **TL (mm)** | 17.09 ±0.15 | 16.87±0.19 | 17.31±0.14 | 17.05±0.18 ^ns^ |
| **Heart (mg)** | 125.7±5.7 | 109.9±1.0 | 213.3±9.9* | 199.0±8.6* |
| **LV (mg)** | 99.1±4.9 | 85.1±1.1 | 164.1±5.8* | 162.9±7.0* |
| **LA (mg)** | 3.9±0.4 | 3.1±0.2 | 16.6±4.1* | 7.7±0.8 |
| **RV (mg)** | 21.0±0.9 | 18.6±0.2 | 30.4±1.8* | 25.5±1.1# |
| **lung (mg)** | 145.5±4.2 | 154.5±19.3 | 259.5±23.8* | 196.6±15.2# |
| **Spleen (mg)** | 84.8±4.8 | 74.5±4.6 | 98.3±4.3 | 96.2±2.7 ^ns^ |
| **Heart/BW (mg/g)** | 4.39±0.10 | 4.26±0.08 | 7.39±0.42* | 6.90±0.30* |
| **LV/BW (mg/g)** | 3.46±0.09 | 3.30±0.06 | 5.68±0.27* | 5.63±0.22* |
| **LA/BW (mg/g)** | 0.13±0.01 | 0.12±0.01 | 0.57±0.14* | 0.27±0.03 |
| **RV/BW (mg/g)** | 0.74±0.02 | 0.72±0.02 | 1.06±0.08* | 0.89±0.04 |
| **Lung/BW (mg/g)** | 5.13±0.06 | 5.94±0.58 | 9.15±1.01* | 6.35±0.33# |
| **Spleen/BW (mg/g)** | 2.94±0.11 | 2.88±0.10 | 3.40±0.11* | 3.34±0.09 |
| **Heart/TL (mg/mm)** | 7.39±0.38 | 6.51±0.05 | 12.34±0.58* | 11.73±0.53* |
| **LA/TL (mg/mm)** | 0.23±0.02 | 0.18±0.01 | 0.95±0.23* | 0.46±0.05 |
| **Ventricles/TL (mg/mm)** | 7.07±0.37 | 6.15±0.06 | 11.26±0.44* | 11.11±0.49* |
| **Spleen/TL (mg/mm)** | 5.01±0.31 | 4.41±0.23 | 5.71±0.25 | 5.66±0.17 ^ns^ |

**Supplementary Table S3. Anatomic data of 4 experimental groups in mice.**

Data are presented as mean ± SEM. BW, body weight; TL, tibial length; LV, left ventricle; LA, left atrium; RV, right ventricle. *p<0.05 *vs.* corresponding sham mice, #p<0.05 *vs.* TAC + isotype mice, ns, not significant for all groups.
